# Abnormal Oxidative Stress Responses in Fibroblasts from Preeclampsia Infants

**DOI:** 10.1371/journal.pone.0103110

**Published:** 2014-07-24

**Authors:** Penghua Yang, Aihua Dai, Andrei P. Alexenko, Yajun Liu, Amanda J. Stephens, Laura C. Schulz, Danny J. Schust, R. Michael Roberts, Toshihiko Ezashi

**Affiliations:** 1 Division of Animal Sciences, University of Missouri, Columbia, Missouri, United States of America; 2 Department of Obstetrics, Gynecology, & Women’s Health, University of Missouri, Columbia, Missouri, United States of America; 3 Bond Life Sciences Center, University of Missouri, Columbia, Missouri, United States of America; 4 Department of Biochemistry, University of Missouri, Columbia, Missouri, United States of America; 5 College of Life Science, Yangtze University, Jingzhou, Hubei, China; 6 Shaanxi Centre of Stem Cells Engineering and Technology, Northwest A&F University, Yangling, Shaanxi, China; Univ Mississippi Medical Center, United States of America

## Abstract

**Background:**

Signs of severe oxidative stress are evident in term placentae of infants born to mothers with preeclampsia (PE), but it is unclear whether this is a cause or consequence of the disease. Here fibroblast lines were established from umbilical cords (UC) delivered by mothers who had experienced early onset PE and from controls with the goal of converting these primary cells to induced pluripotent stem cells and ultimately trophoblast. Contrary to expectations, the oxidative stress responses of these non-placental cells from PE infants were more severe than those from controls.

**Methods and Findings:**

Three features suggested that UC-derived fibroblasts from PE infants responded less well to oxidative stressors than controls: 1) While all UC provided outgrowths in 4% O_2_, success was significantly lower for PE cords in 20% O_2_; 2) PE lines established in 4% O_2_ proliferated more slowly than controls when switched to 20% O_2_; 3) PE lines were more susceptible to the pro-oxidants diethylmaleate and tert-butylhydroquinone than control lines, but, unlike controls, were not protected by glutathione. Transcriptome profiling revealed only a few genes differentially regulated between PE lines and controls in 4% O_2_ conditions. However, a more severely stressed phenotype than controls, particularly in the unfolded protein response, was evident when PE lines were switched suddenly to 20% O_2_, thus confirming the greater sensitivity of the PE fibroblasts to acute changes in oxidative stress.

**Conclusions:**

UC fibroblasts derived from PE infants are intrinsically less able to respond to acute oxidative stress than controls, and this phenotype is retained over many cell doublings. Whether the basis of this vulnerability is genetic or epigenetic and how it pertains to trophoblast development remains unclear, but this finding may provide a clue to the basis of the early onset, usually severe, form of PE.

## Introduction

Preeclampsia (PE) is a relatively common disease of pregnancy, affecting ∼5% of pregnancies, with major racial, social and geographical disparities [1]–[3]. It is characterized by onset of hypertension and proteinuria, with symptoms appearing in the second half of pregnancy [4]. The more serious, early onset form of the disease can manifest as soon as 20 weeks after conception, but, more commonly, symptoms appear nearer to term and are usually less life threatening [5]. The underlying cause of PE is usually considered to be insufficient perfusion of maternal blood through the placenta in the second half of pregnancy and is presumed to stem from a failure of the uterine spiral arteries to become appropriately remodeled by invading extravillous trophoblast (EVT) during the first trimester well before symptoms become evident [6]. No method exists for diagnosing PE at these early stages of pregnancy, making any study of the pathophysiology of the disease difficult, especially in the delivered placenta when proliferative capacity of trophoblast (TB) is largely exhausted and the generation of additional EVT has ended. Thus, phenotypic abnormalities in EVT should be evident in the first trimester TB when PE is initiated but cannot be recognized. However, the causative underpinnings of the disease may not persist until the time when the baby is delivered, when TB invasion has ceased.

Why growth of EVT and possibly even villous TB [7] in the first trimester is impeded remains controversial, but low or erratic perfusion rates at later stages of the pregnancy probably lead to oxidative stress throughout the placenta with accompanying free radical damage to DNA and cellular structures arising from irregular spells of reperfusion with oxygenated blood [8]–[12]. Together, these insults have been proposed to increase cell turnover and shedding of debris from the syncytioTB, as well as production of a range of compounds, including cytokines and anti-angiogenic agents, that may cause inflammatory responses in the mother beyond those experienced in a normal pregnancy [9], [13]–[15]. An additional difficulty in studying PE is that it is a complex disease, often with familial clustering but with no clear inheritance patterns [16]–[18], although the early onset forms have been inferred to have a stronger fetal/paternal genetic component than the later onset forms where PE may be more associated with susceptibilities of the maternal cardiovascular system to the presence of a placenta [19].

Our long term goal is to create human induced pluripotent stem cells (iPSC) [20]–[22] from UC cells from infants born to mothers with early onset PE, and, once lines have been established, convert these cells to TB representing an early stage of the pregnancy [23]–[25]. In this paper, we describe the first steps in achieving this end, namely the establishment of primary mesenchymal cells from explants of UC. In doing so, we discovered that the primary fibroblast cultures derived from patients with PE were unusually sensitive to oxidative stressors compared to controls (CTL) and that this phenotypic difference was maintained over multiple cell doublings. We hypothesized that fibroblasts derived from the infants with the early onset form of PE might be either genetically distinct from CTL fibroblasts or else had acquired a stable epigenetic memory that rendered them less able than the CTL to endure a switch to high O_2_.

## Methods

### Human umbilical cord tissue collection

The human umbilical cords (UC) were collected from 17 infants whose mothers suffered early onset PE and from 10 controls (CTL) at Women’s and Children’s Hospital (University of Missouri, Columbia, MO) between 2010 and 2013 (Table S1 in File S2). The diagnostic criteria for PE were as designated by the American Congress of Obstetricians and Gynecologists [26]. Briefly, these criteria included women with new onset severe hypertension, proteinuria or severe systemic signs (elevated liver enzymes, low platelets, hemolysis) or symptoms (unremitting headache, visual changes, right upper quadrant pain) requiring very preterm delivery. Small (approximately 2 cm) segments of aseptically collected umbilical cord were immediately placed in 20 ml low glucose (LG, 5.6 mM) DMEM medium supplemented with Primocin (InvivoGen, San Diego, CA) to minimize microbial contamination and transferred to the cell culture lab for processing. The University of Missouri Health Sciences Institutional Review Board (IRB) considers placental tissues including UC to be discarded tissues and waived the need for written consent. The UC tissue collection project (#1201132) has been approved by the University of Missouri Health Sciences IRB. This protocol for the collection of de-identified tissues allowed us to also record the gestational age at delivery and reason for delivery. A separate protocol (protocol #1209459) was approved by the University of Missouri Health Sciences IRB that allowed us to record use of antenatal steroids prior to delivery, gender of the delivered infant(s), and mode of delivery. The IRB did not approve collection of any additional data on deliveries either for severe preeclampsia or from those of gestational age-matched and full-term controls.

### Outgrowth of human umbilical cord (UC) fibroblasts under different conditions

**Figure 1:**
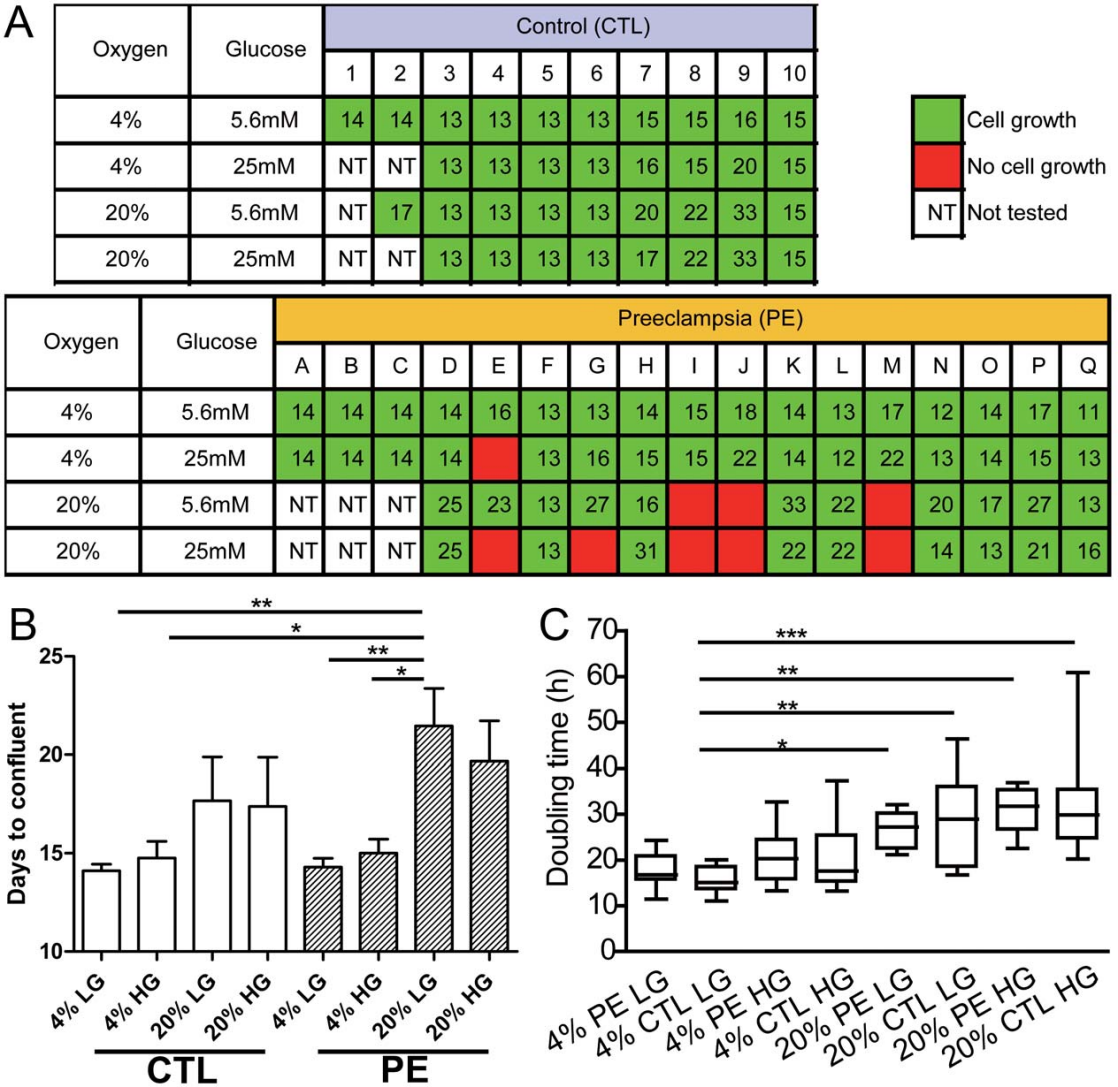
An examination of the effects of two different oxygen atmospheres (4% and 20%) and two glucose concentrations (5.6 mM and 25 mM) on proliferation of human UC fibroblasts derived from CTL babies and babies born to mothers with severe PE. (A) Days required for outgrowths to reach confluence after placing minced UC explants into culture medium on a gelatin substratum. Green boxes indicate cultures where outgrowths were observed. Red boxes show conditions where there were no outgrowths. Open boxes (NT, not tested) are where those particular conditions were not applied. Numbers within boxes are days required to reach confluence within the culture wells. Cultures 1–10 and A–Q were derived from the babies listed in Table S1 in File S2. (B) Summary of numerical data on days to reach confluence derived from Fig. 1A for the four different conditions employed to generate outgrowth of fibroblasts from the different UC samples. PE 20% O_2_ HG bar is represented without data from E, G, I, J and M because cultures were not established under these conditions (as shown Fig. 1A). Similarly, data from I, J and M samples are not included for the PE 20% O_2_ LG bar. PE 4% O_2_ HG lacks the data from line E. Data analysis was by ANOVA (*P≤0.05; **P≤0.01). (C) Box & Whisker plot showing the population doubling times at p 3 for UC fibroblasts cultured under the four different conditions. The fibroblasts, maintained under these conditions (HG/LG medium, high/low O_2_), were dispersed into 12-well culture plates at a density of 2.0×10^4^ cells/well in 1 ml of medium. Cell numbers were measured after 24, 48, 72 and 96 h for each primary cell line. These data were used to calculate population doubling times (DT). Data analysis was by ANOVA (*P≤0.05; **P≤0.01; ***P≤0.001).

Human umbilical cord (UC) tissues were washed at least twice with phosphate buffered saline (PBS) to remove blood cells and cut into two pieces. One was placed in high glucose (HG, 25 mM), the other in low glucose (LG, 5.6 mM) DMEM medium. Tissues were minced into 1–2 mm^3^ fragments with scissors and placed into a 48-well plate (one small piece per well; 12 wells for HG and 12 wells LG medium, respectively) that had been coated with 0.1% gelatin in HG/LG medium containing 10% FBS, 1% NEAA, 2 mM glutamine, 0.1 mM 2-mercaptoethanol and 4 ng/ml FGF2, and cultured at 37 C in an atmosphere of either 4% O_2_/5% CO_2_/91% N_2_ or 5% CO_2_/air (20% O_2_) to generate outgrowths of adherent cells [27], [28]. The cultures were kept undisturbed for the first 5–7 days and supplemented with Primocin to reduce risk of bacterial and fungal growth in the primary culture. The medium without Primocin or antibiotics was refreshed every two days thereafter until the adherent cells from the tissue fragments formed outgrowths, which usually began to appear at the periphery of the minced tissues after about a week in all cultures in 4% O_2_ and in only a select number of cultures in 20% O_2_. Once a close-to-confluent monolayer of fibroblasts was achieved, the cells were sub-cultured from the 48-well plates into T25 flasks by using TrypLE (Invitrogen, Carlsbad, CA) proteinase. When the cells had reached to ∼90% confluence in the T25 flask, the cells (passage 1: p 1) were frozen by adding 2x Profreeze, a chemically defined freeze medium (LONZA, Basel, Switzerland), to generate cell stocks in two cryovials per T25 flask. The number of days from the explant initiation was recorded as shown in Fig. 1.

### Routine growth and sub-culture of cells

The UC fibroblast cultures were maintained and expanded in the same manner as described above with fresh medium added every two days. When a culture reached 80–90% confluent, cells were detached with TrypLE and split at a ratio of 1∶4 into new T-25 flasks coated with 0.1% gelatin. Cell stocks were prepared at every passage by the method described above.

### Sensitivities of UC fibroblasts to high oxygen and high glucose

The primary UC fibroblasts maintained under the different conditions were passaged into individual wells of 12-well plates at an initial cell density of 2.0×10^4^ cells/well at p 3 and cell numbers counted at 24, 48, 72 and 96 h after dispersing with TrypLE and counting cell numbers with a TC10 Automated Cell Counter (Bio-Rad, Hercules, CA). Doubling time (DT) was calculated by using the formula of DT = (tn_2_−tn_1_)×log(2)/log(N_tn2_/N_tn1_) where tn_1_ is the earlier sampling time (usually 24, 48, 72 h) and tn_2_ the later sampling time (usually 48, 72 and 96 h), N_tn1_ is cell number at tn_1_. Averaged DT was obtained from nine DT measurements per cell line, i.e., three (tn_2_−tn_1_) points of (48–24), (72–48), (96–72) in three culture replicates.

### The effects of switching cells from a low to a high O_2_ atmosphere

In these experiments, four PE cell lines (G, I, J, M) that could be established and stably maintained under 4% O_2_ condition but that failed to establish outgrowths in 20% O_2_ were grouped as O_2_ sensitive (**O_2_-s**). Three other PE lines (D, H, L) that had formed outgrowths under both conditions were grouped as O_2_ non-sensitive (**O_2_-n**). Both sensitive and non-sensitive groups were compared with four control cell lines (7, 8, 9, 10). All cell lines had been maintained continuously in 4% O_2_/LG conditions. At p 3 the cells were subcultured into 6-well plates at an initial density of 40,000/well and cultured further under either 20% or 4% O_2_ conditions. Cell numbers at respective time points were counted as above.

### Treatment of cells with diethyl maleate (DEM) and tert-butylhydroquinone (tBHQ)

Induction of oxidative stress was performed according to the method of Shibuya et al. [29]. The UC fibroblasts were passaged into wells (area 0.32 cm^2^) of a 96 well plate at a density of 1.0×10^4^/cm^2^ for 24 h under 4% O_2_ in 0.1 ml of medium containing DEM (Sigma, #W505005) at concentrations ranging from 0.1 mM to 1.0 mM. tBHQ (Sigma #112941) was tested similarly over a concentration range of 0.02 to 0.2 mM [29]. To terminate the experiment, cells were washed 3x with PBS, fixed with buffered 10% formalin solution (pH 7.2), and then stained with 0.1% crystal violet in H_2_O. After cells were rinsed, dye was eluted with 10% ethanol in 0.1 M sodium citrate. Finally, the optical density of the dye solution (OD) was measured at 595 nm (Synergy HT plate reader, Bio-Tek, Instruments Inc. Winooski, VT). The cell survival rates were calculated by using the formula (OD_DEM_ −OD_Blank_)/(OD_control_ −OD_Blank_).

### Flow cytometric analysis of cell cycle distribution

UC fibroblasts (2×10^4^ cells) dissociated with TrypLE were resuspended in PBS (1 ml) and then fixed in cooled 70% ethanol (2.5 ml) at 4°C for 30 min. The cells were incubated in 0.5 ml PBS solution containing 1 mg/ml RNase A, 20 µg/ml propidium iodide (Fisher Pittsburgh, PA), and 0.05% Triton X-100 (Fisher) at 37°C for 40 min. They were then resuspended in 0.5 ml PBS for flow cytometry. Cell cycle analysis by DNA content was performed in triplicate with independently prepared samples. For each cell population, at least 10,000 cells were analyzed in the Accuri C6 Flow Cytometer (BD Biosciences), and the proportion in G_0_/G_1_, S, and G_2_/M phases calculated with FlowJo 7.6.5 software (Tree Star Inc. Ashland, OR).

### Reduced glutathione (GSH) concentrations in UC fibroblasts maintained under different O_2_ conditions

The determination of intracellular thiol concentrations of 6 PE cell lines (G, K, L, N, O, P) and 6 CTL cell lines (3, 6, 7, 8, 9, 10) after culture in 20% and 4% O_2_ was performed with a Fluoro Thiol detection kit (Cell Technology Inc., Mountain View CA). The kit contains a fluorescein dye (2, 4-dinitrobenzenesulfonyl 2′, 7′-dimethylfluorescein) that reacts with thiols at neutral pH, cell lysis buffer, and GSH standards. Cell lysates were prepared from early passage (p 1 or p 2) UC fibroblasts (4×10^5^ cells/160 µl) by using the cell lysis buffer supplied with the kit. Aliquots (n = 3) of 50 µl cell lysate were placed into individual wells of an opaque (black) 96-well plate, mixed with 50 µl freshly constituted fluorescein dye solution, and incubated at room temperature in the dark for 10 min. Readings were obtained at an excitation of 488 nm and emission of 515–530 nm with a Synergy HT plate reader.

### Microarray analysis of cells grown under 4% O_2_ and 20% O_2_

RNA was isolated from early passages (<p 5) of UC fibroblast lines (15 PE and 9 CTL lines, Table S1 in File S2) when they reached ∼90% confluence in T25 flasks under 4% O_2_ conditions. In order to collect RNA from cells under 20% O_2_, cell lines at either p 4, 5, or 6 were switched from 4% O_2_ conditions to 20% O_2_ conditions when they were approximately 20% confluent. When they reached ∼90% confluence (generally 3–4 days in 20% O_2_ conditions and 2 days in 4% O_2_), medium was removed and RNA STAT60 (1 ml; Tel-Test, Friendswood, TX) immediately added to each T25 flask. The RNA was further purified according to the manufacturer’s instructions. RNA samples were submitted to the University of Texas Southwestern Medical Center Microarray Core Facility (https://microarray.swmed.edu/), and microarray analysis performed with Illumina HumanHT-12 v4 expression BeadChips. Raw intensity data were background subtracted by using BeadStudio software and analyzed further by GeneSpring 12.6 software (Agilent Technologies Inc., Santa Clara CA), according to the advanced workflow protocol: percentile shift and filter by flags (detected). An unpaired t-test analysis was applied to identify differentially expressed genes (P≤0.05) whose fold change between PE and CTL was ±1.5 or greater. Differentially expressed gene lists were subjected to gene ontology analysis by using DAVID (http://david.abcc.ncifcrf.gov/) with default parameters. Principal component analysis, which provides an unsupervised clustering and visualization approach for analyzing data derived from gene expression arrays, was applied to the 4% O_2_ cultures of 14 PE and 9 CTL lines and the 20% O_2_ cultures of 15 PE and 9 CTL lines, i.e. 47 samples in total (Table S1 in File S2). This analysis employed the entire 47159 probes on the Beadchip to look for underlying cluster structures. The microarray data have been deposited in the Gene Expression Omnibus (GEO) database, www.ncbi.nlm.nih.gov/geo (accession no: GSE54400).

### Quantitative RT-PCR

RNA was extracted from CTL and PE fibroblasts in STAT60 containing 1-bromo-3-chloro-propane (Sigma-Aldrich) and purified by using the TURBO-DNA-free kit (Ambion). It was reverse transcribed with the SuperScript VILO cDNA Synthesis kit (Invitrogen). Primers were designed and verified against the GenBank database by using Primer-BLAST online software (National Center for Biotechnology Information, www.ncbi.nlm.nih.gov) and were synthesized by Integrated DNA Technologies. Accession numbers, primer sequences, length, and melting temperatures (Tm) for all tested genes and predicted sizes of the amplified fragments are summarized in Table S9 in File S2. Quantitative RT-PCR (qRT-PCR) was performed with SYBR GreenER qPCR SuperMix Universal (Invitrogen) on an ABI 7500 Real-Time PCR System (Applied Biosystems). GAPDH was selected as the reference gene. PCR conditions were as follows: 50°C for 2 min; 95°C for 10 min; followed by 40 amplification cycles (95°C for 15 s and 60°C for 60 s). Reactions were terminated by an elongation and data acquisition step at 75°C for 45 s. *GAPDH* primers demonstrated reliable expression of the reference gene under these conditions. Values were normalized to *GAPDH* values obtained in the same run. The comparative threshold cycle (CT) method was used to calculate relative values [30] as described elsewhere [31].

### Statistical analysis

Statistical analyses were performed by using GraphPad Prism 5 software (GraphPad Software, Inc., La Jolla, CA). Data were subjected to one-way ANOVA followed by Tukey’s test. Where appropriate, a nonparametric Student’s t-test was employed for pairwise comparisons of data from individual groups of cells. Values of P≤0.05 were considered to support the conclusion that differences were significant.

## Results

### Patient characteristics

Segments of UCs were derived from 17 infants from mothers (one twin pregnancy and 15 singletons) who had experienced severe PE (Table S1 in File S2). Lengths of gestation ranged from approximately 27 weeks (L & K) to 34 weeks (E & M). All PE patients were delivered by Cesarean section due to maternal or fetal indications, and all but two (B & E) had received betamethasone therapy to minimize neonatal respiratory disease. Two (I & J) were monozygotic twins. In what appears to be chance, only two (A & P) of the 17 PE infants were females. Two mothers had been diagnosed with HELLP syndrome (A & E).

Cords were also derived from 10 CTL neonates (5 males and 5 females), again in a non-discriminating manner. Gestational ages ranged from 33 weeks to 40 weeks, but the majority was comprised of normal term infants, four of which were delivered by Cesarean section. Two CTL infants (#7, delivered at 34 weeks, and #8, delivered at 33 weeks) were preterm due to premature rupture of membranes. Only the former had been exposed to betamethasone.

### Outgrowth of UC mesenchyme under different culture conditions

In the initial experiments performed with UC from three infants born to mothers with PE (cultures A, B, & C) and to two CTL (cultures 1 & 2), no attempt was made to make a systematic study of the culture requirements for successful outgrowth (Fig. 1A). In each instance, however, primary cultures were established successfully under 4% O_2_ conditions with DMEM medium containing both low (5.6 mM; LG) and high glucose (25 mM; HG). In subsequent studies, either physiological (low, 4%) or atmospheric (high, 20%) O_2_ conditions were used to establish cultures. Similarly, either a LG or HG culture medium was employed. The two variables, O_2_ and glucose concentration, were tested in a 2×2 factorial design to examine whether these parameters influenced outgrowth from the solid tissue.

As illustrated in Fig. 1A, no difficulties were encountered with CTL tissues under any of the four conditions tested, although there were indications that high O_2_, but not HG, introduced more variability and extended the time required to achieve a confluent culture (Fig. 1B). The picture was different with the explants from PE infants (Fig. 1A; Table 1). With the exception of explant culture E, where there was no growth in HG in either O_2_ condition, primary cultures were successfully established from 16 of the 17 PE infants with a 4% O_2_ gas atmosphere under both HG and LG conditions. However, only 61.5% (8/13) of the PE cords provided primary cultures under 20% O_2_/HG conditions. Success improved when the glucose concentration was lowered to 5.6 mM, when 10 of 13 cultures became established. Notably, however, three explants (I, J, M) failed to provide outgrowths under high O_2_ at both glucose concentrations. Moreover, high O_2_, whatever the glucose concentration, significantly lengthened the time to establish confluent cultures for PE lines examined (means for low and high O_2_ conditions 14.64±0.42 days and 20.65±1.38 days, respectively, p<0.0001) (Fig. 1B). Together, these data, which indicate that PE cultures had a reduced ability to form outgrowths and that outgrowths that did form required a longer time to reach confluence under high O_2_, suggested that outgrowths of UC from PE infants were more sensitive to O_2_ stress than those from CTL.

**Table 1:** Effect of a difference in oxygen atmosphere and glucose concentration in the culture medium on the ability of umbilical cord explants to form outgrowths.

	PE		CTL	
	UC	Outgrowth	UC	Outgrowth
	(n)	(n)	(n)	(n)
4% **O_2_** LG	17	17	10	10
4% **O_2_** HG	17	16	8	8
20% **O_2_** LG	13	10	9	9
20% **O_2_** HG	13	8*	8	8

*Outgrowth number significantly reduced (P<0.05).

### The population doubling time (DT) of the primary fibroblast lines

DT values were measured at p 3 in cell lines that had been maintained under the conditions that had been used for their derivation (Fig. 1C). At 4% O_2_, the PE cell lines proliferated at about the same rate as CTL cell lines in both LG and HG medium (Fig. 1C), although the variances and range of values were higher under HG than LG. Both the PE and CTL cultures proliferated significantly more slowly in high than in low O_2_ (Fig. 1C), but again values for PE and CTL cell lines were not distinguishable. Cell doubling times were also not influenced by glucose concentration. Together these data suggest that, even though it is more difficult to establish UC fibroblast lines from PE infants than from CTL infants under 20% O_2_, the cells adapt sufficiently under these conditions during the outgrowth period that their doubling times become quite similar.

### Cell morphologies and growth of UC fibroblasts in response to a transfer from a low to high O_2_ atmosphere

**Figure 2:**
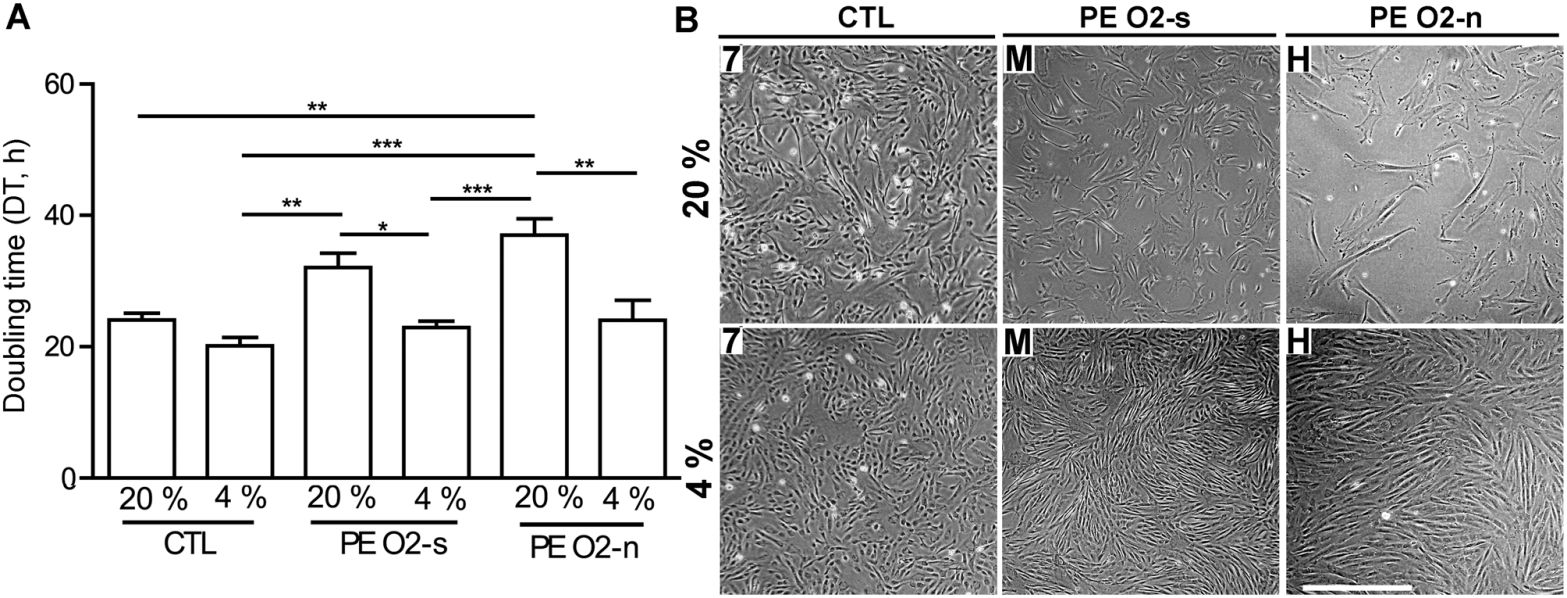
Effect of switching UC fibroblasts from 4% O_2_ to 20% O_2_ conditions on cell proliferation. (A) Doubling time of cells after switching to 20% O_2_. Cell lines were in three groups: CTL (#7, 8, 9, 10), PE O_2_ sensitive (O_2_-s; G, I, J, M) that had not formed outgrowths under 20% O_2_, and PE O_2_ non-sensitive (O_2_-n; D, H, L) that had formed outgrowths under 20% O_2_. For this experiment, p 1 frozen cells established under 4% O_2_ conditions were thawed and cultured in T-25 flasks under 4% O_2_. When these p 2 cells were ∼90% confluent, they were transferred to 6-well plates (20,000/well in DMEM-LG medium under either 20% or 4% O_2_ conditions. Cell numbers were assessed at 24, 48, 72, and 96 h after sub-culture to calculate population doubling times. Values are means ± SEM (CTL, n = 4, PE O_2_-s, n = 4, PE O_2_-n, n = 3). The statistical analysis was conducted by ANOVA (*P≤0.05; **P≤0.01, ***P≤0.001). (B) Cell morphologies of selected PE and CTL cell lines after switching the cells from 4% to 20% O_2_ at p 3 and culturing for a further 5 days (top panels). The controls (lower panels) were continuously maintained under 4% O_2_ for 5 days. The experiment was identical to that in A, and images shown are of the CTL line #7, PE line M (an O_2_-s line) and PE line H (an O_2_-n line). Bar, 0.5 mm. Images of other PE & CTL lines from this experiment are shown in Fig. S1 in File S1. The slower growth under 20% O_2_ conditions required five days of culture to provide sufficient cells in the field of view to represent a typical population.

In this experiment, the fibroblast lines employed had been established under low (4%) O_2_, maintained under these conditions for two passages, and then either cultured under the same conditions or transferred to 20% O_2_ at p 3. First, we examined the population doubling times of four CTL (7, 8, 9, & 10) and two sets of PE cultures to determine how cell growth rates were affected by a switch in O_2_ atmosphere. The first of these were cell lines (G, I, J, & M; **O_2_-s**) that had failed to establish outgrowth at 20% O_2_ in at least one of conditions. The second set of PE cultures (D, H, & L; **O_2_-n**) had been able to form outgrowths from the UC explants under 20% O_2_, as well as under 4% O_2_ and were hypothesized to be less sensitive to O_2_ than the first group (Fig. 1A). All three sets of cultures demonstrated similar doubling times (of about 20 h) under the lower O_2_ atmosphere (Fig. 2A). The PE groups (**O_2_-s** & **O_2_-n**) exhibited similar doubling times to each other under 20% O_2_, but both values were significantly greater than those noted for CTL maintained under identical culture conditions. In other words, the switch from low to high O_2_ conditions was more severely deleterious for the PE lines than for the CTL lines.

We then sought to determine whether the differences in growth rates were correlated with changes in cell morphologies. The same cultures as above were investigated All retained similar fibroblast-like morphologies at p 3 when cultured on DMEM with 5.6 mM glucose under a 4% O_2_ atmosphere (Fig. 2B, Fig. S1 in File S1). This normal fibroblast morphology was maintained without discernible change in all the CTL and PE cultures over five passages, which is equivalent to over 20 population doublings. Some cultures have been passaged more than 10 times without an apparent loss of viability. By contrast, abnormal morphologies in the form of long, spindle-shaped cells were evident in the eight PE cultures immediately after transfer to 20% O_2_ at p 3 (Fig. 2B, Fig. S1 in File S1). The effects of the switch to high O_2_ were less marked in CTL than in PE cultures at p 3 but did begin to manifest after further passage. Thus, both types of cell are sensitive to high O_2_, but the morphological changes occur earlier in the PE cultures. Together, these data provide further evidence that PE cultures, as a whole, are more sensitive than CTL when they are switched from 4% O_2_ to 20% O_2_ conditions.

### Determination of glutathione (GSH) content of cells under different culture conditions

**Figure 3:**
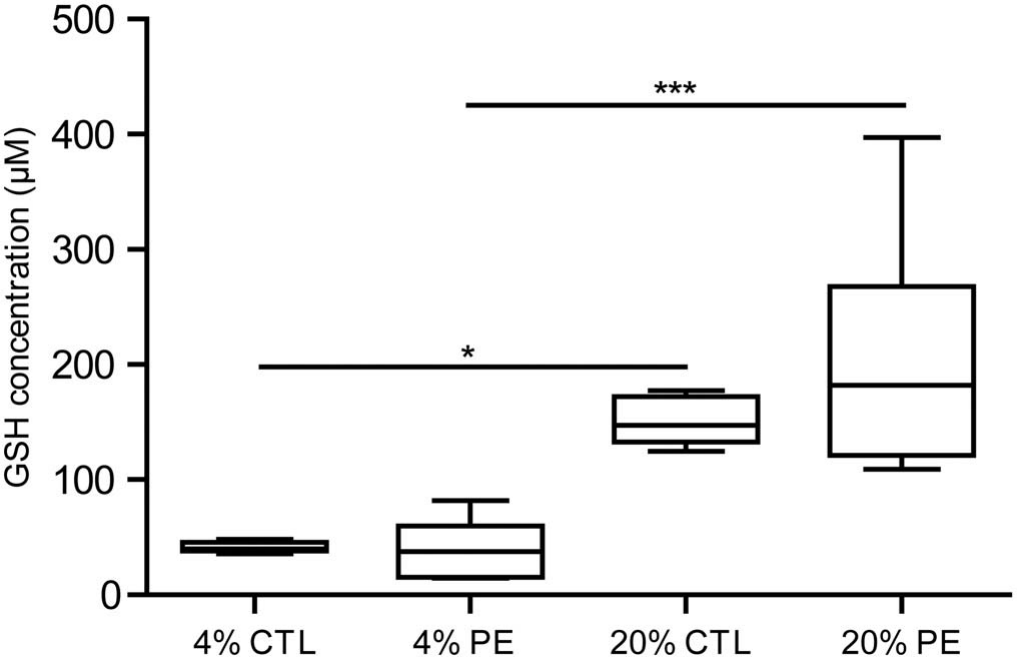
Box and whisker plot showing the concentration of reduced glutathione in UC fibroblasts maintained under different O_2_ conditions. Six PE lines (G, K, L, N, O, P) and six control lines (#3, 6, 7, 8, 9, 10) at p 2 that had been cultured under either 4% O_2_ or 20% O_2_ conditions until they reached approximately 90% confluence, were dispersed by using TrypLE. Aliquots equivalent to 4×10^5^/cells were lysed in 160 µl of lysis buffer, and 50 µl aliquots assayed for total GSH content. Values shown are concentrations in the lysis buffer. The statistical analysis was conducted by ANOVA (*P≤0.05, ***P≤0.001).

Healthy cells and tissues possess mechanisms for maintaining an optimal redox state, one of which depends on the relative concentrations of oxidized and reduced glutathione. To determine whether or not the greater sensitivity of PE cells to high O_2_ might involve a defect in this redox control system, we measured the reduced glutathione concentration in six PE cultures and in six CTL between p 1 and p 2 after they had been grown under either 4% or 20% O_2_. GSH concentrations were normalized to provide values for 1.25×10^5^ cells/culture (Fig. 3). There was no difference in intracellular glutathione concentrations between PE and CTL cultures under either high or low O_2_ conditions, but levels were higher for both under high O_2_ compared to cells cultured under low O_2_ (PE, P<0.05; CTL, P<0.001, respectively).

### Effect of pro-oxidant chemicals on cell death and proliferation of UC fibroblast cells

**Figure 4:**
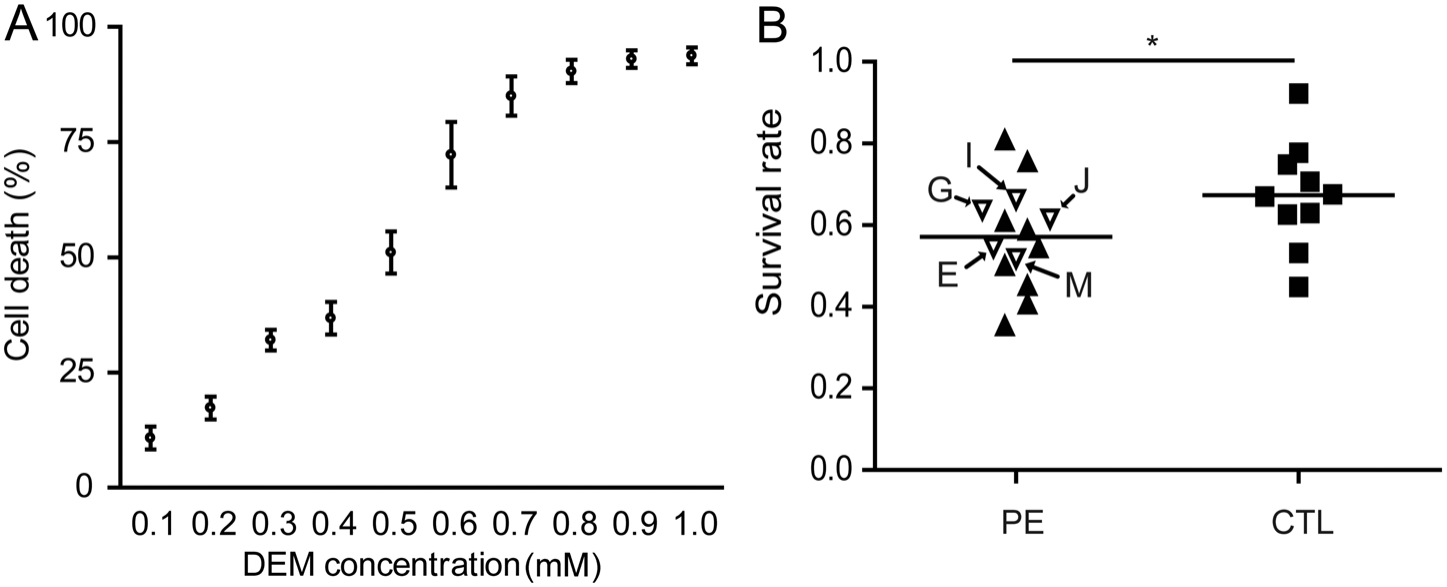
Effect of diethylmaleimide (DEM) on growth of UC fibroblasts. (A) Dose response to increasing concentrations of DEM. Fibroblasts (4 PE, L, M, N, O; 4 CTL, #6, 7, 8, 9) were sub-cultured into 96-well plates at p 3 at a density of 3,200 cells/well in LG/4% O_2_ conditions for 24 h. They were then exposed to DEM (final concentrations 0.1 mM to 1.0 mM) for a further 24 h under the same culture conditions. The washed cells were fixed and stained with 0.1% crystal violet, the dye extracted in 10% ethanol/0.1 M sodium citrate, and absorbance (OD) measured at 595 nm. Values are presented as (OD_control_−OD_DEM_)/OD_control._ (B) Relative abilities of PE and CTL fibroblasts to survive 0.4 mM DEM for 24 h. The experimental design was that in Fig. 4A, but utilized a single concentration of DEM and 14 PE and 10 CTL lines. The open triangles indicate the five PE (O_2_-s; E, G, I, J, M) lines that failed to form outgrowths in 20% O_2_ (*P≤0.05).

We hypothesized that the sensitivities of the PE cultures to high O_2_ might be paralleled in their responses to pro-oxidant chemicals, such as DEM and tBHQ that deplete intracellular glutathione. In initial experiments, we established that a concentration of DEM above 0.7 mM was sufficient to cause most cells in the cultures grown on low glucose and in 4% O_2_ to detach and presumably die within 24 h (Fig. S2 in File S1). This acutely toxic effect at the higher concentrations appeared to be similar for both PE and CTL cultures. We then tested four CTL and four PE lines to concentrations of DEM ranging from 0.1 mM to 1 mM to determine an approximate concentration that permitted survival over a 24 h exposure (Fig. 4A). Although the dose responses to DEM varied across fibroblast lines, concentrations of DEM below 0.5 mM appeared to be appropriate to judge relative toxicity of the chemical.

We next examined the responses of cell lines to 0.4 mM DEM under 4% O_2_ conditions. While the survival rate of CTL cells (n = 10) was 67.4±13.1%, that of the PE cultures (n = 14) was 57.1±12.6%, indicating that the PE-cell lines were marginally more susceptible to acute DEM toxicity than CTL (P<0.05) (Fig. 4B). There was no suggestion that the lines that had failed to form outgrowths under high O_2_ conditions (Fig. 4B: cultures E, G, I, J, M: arrowed) were any more sensitive to DEM than the other PE cultures where it had been possible to generate fibroblast outgrowths. Similarly, after initially testing tBHQ over the concentration range 0.02 mM to 0.2 mM, we selected 0.16 mM as a an oxidative stressor for cells grown under 4% O_2_ conditions. Cell viability again varied across lines, but the four PE lines again showed lower viability (P<0.05) than the CTL lines (Fig. S3A & B in File S1).

**Figure 5:**
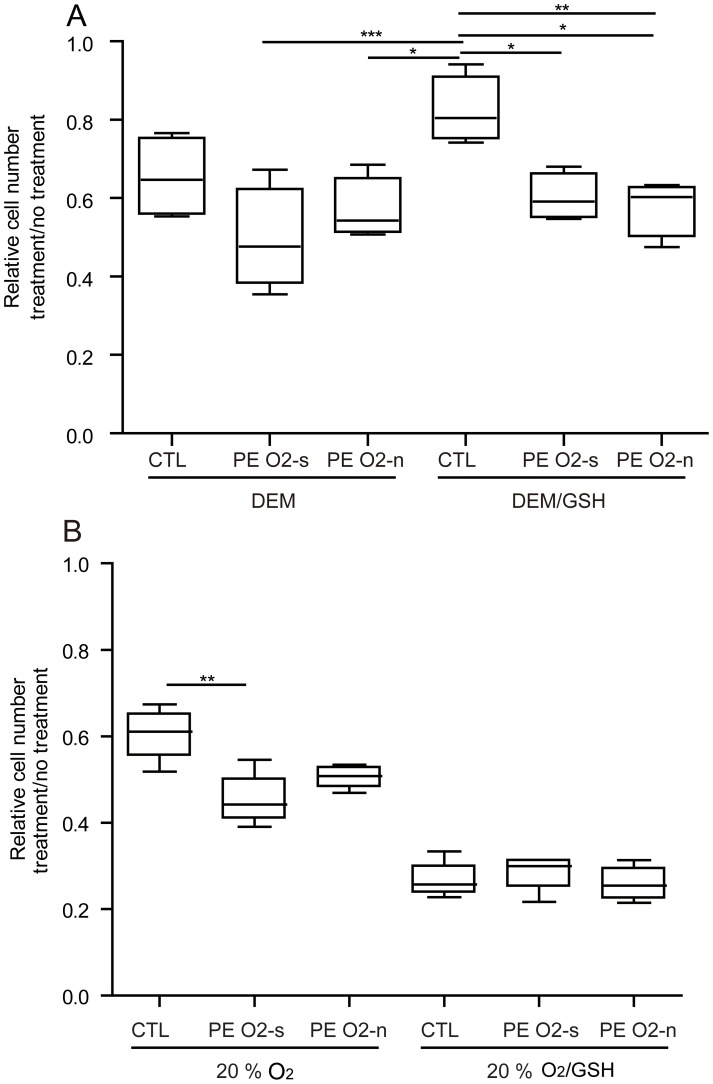
Box and whisker plots showing the effect of 0.1 **mM DEM in presence and absence of 10 mM GSH on proliferation of PE and CTL UC fibroblasts under 4% (A) and 20% O_2_ (B) conditions.** Each line had been maintained under 4% O_2_ conditions for five passages prior to the experiment. Cell lines were in three groups: CTL (#7, 8, 9, 10), PE O_2_ sensitive (O_2_-s; G, I, J, M) that had not formed outgrowths under 20% O_2_, PE O_2_ non-sensitive (O_2_-n; D, H, L) that had formed outgrowths under 20% O_2._ At p 6, 20,000 cells were dispensed into wells of 6-well plates to begin the treatments. Cells were counted at 72 h. Data are presented as cell number after treatment/cell number with no treatment (*P≤0.05; **P≤0.01; ***P≤0.001).

Next, we determined whether lower concentrations of DEM (less than 0.1 mM) that were not immediately toxic influenced cell proliferation rates over a 96 h period under 4% O_2_. Cell detachment was minimal at all of the DEM concentrations (40, 60, 80, 100 µM) employed (legend of Fig. S4 in File S1), but proliferation rate was significantly slowed (Fig. S4 in File S1). A concentration of 100 µM DEM under 4% O_2_ conditions mimicked the growth curve observed under 20% O_2_ in absence of DEM and reduced the number of cells in s-phase by about half (Fig. S5 in File S1). These conditions (100 µM DEM; 4% O_2_) were then used to compare relative growth sensitivities of PE and CTL cultures. Although 100 µM DEM tended to reduce growth rates of the CTL cultures to a lesser degree than it did in both groups of PE cultures (Fig. 5A), the differences were not statistically significant. However, the presence of reduced glutathione (10 mM) in the medium was largely able to reverse the DEM effects on CTL cultures (P<0.05), but failed to rescue either group of PE cultures (P<0.01) (Fig. 5B).

Under 20% O_2_, 100 µM DEM reduced growth rate of both the CTL and PE cultures to about the same extent as it did under 4% O_2_ conditions (Fig. 5B), but the presence of glutathione worsened rather than reversed the DEM effects in all three groups, including the CTL.

### Effect of gestational age on response of PE and CTL fibroblasts to oxidative stressors

While all the UC from PE infants were obtained from premature births, the majority of CTL cultures were derived from normal term births. To address whether gestational age played an independent role in initiating the distinct cellular phenotypes of PE and CTL fibroblasts, the responses of four selected PE cases (C, D, F, M: mean week of birth 32.89±0.29) and two premature controls (#7 & 8: mean week of birth 33.50±0.50) that did not differ significantly in gestational age (P = 0.315) were compared. The parameters assessed were cell doubling time after being switched from 4% to 20% O_2_ (Fig. S6A in File S1) and 24 h survival rate of the cells after addition of 0.4 mM DEM (Fig. S6B in File S1). The data confirmed that the PE lines were more sensitive to oxidative stress than CTL, with the caveat that the number of appropriate age CTL lines available was limited.

### Comparative transcriptome analysis

In view of the fact that PE and CTL lines differed in sensitivity to oxidative stressors, we hypothesized that the two kinds of cell might exhibit differences in gene expression and that these differences could be exacerbated under stressful conditions. Accordingly, fibroblast cell lines that had been cultured under 4% O_2_ (non-stressed conditions) were either continued under these non-stressful conditions or transferred to 20% O_2_ (stressed) conditions until the cells were near confluent (approximately d 4). RNA analyses were performed on Illumina HumanHT-12v4 bead Chips. A principal component analysis of the normalized microarray data from the two sets of cells, i.e. PE and CTL showed that 20% O_2_ and 4% O_2_ samples formed separate, non-overlapping clusters as a result of significant differences in gene expression under the two growth conditions.

**Figure 6:**
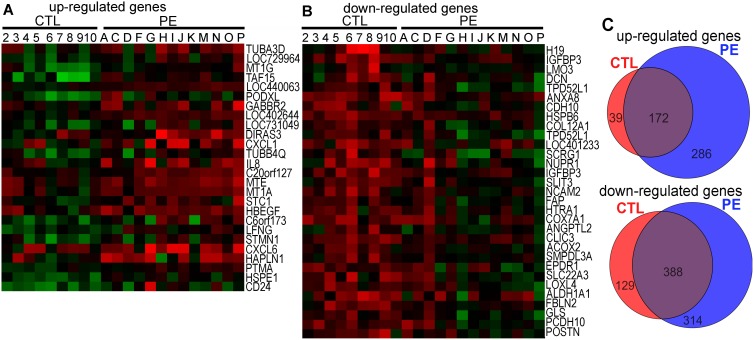
Microarray analysis of UC fibroblasts under 4% O_2_ conditions. (A) Heatmap of up-regulated, differentially expressed genes in PE cells versus controls. (B) Heatmap of down-regulated, differentially expressed, genes in PE *versus* control in 4% O_2_ condition. The cell lines are from the patients listed in Table S1 in File S2. In these analyses, genes associated with the Y-chromosome were not considered because of the strong male bias in the PE cell lines. None of the genes shown in Figs. 6A & B showed any sexual dimorphism in expression. (C) Venn diagrams summarizing the numbers of regulated genes when cells were switched from 4% to 20% O_2_ culture conditions. Upper panel, up-regulated genes (Red, 39 genes up-regulated only in CTL; Blue, 286 genes up-regulated only in PE; Violet, 172 genes up-regulated in both CTL and PE lines); Lower panel, down-regulated genes (Red, 129 genes down-regulated only in CTL; Blue, 314 genes down-regulated only in PE; Violet, 388 genes down-regulated in both CTL and PE lines).

Next, we analyzed the data for differences in gene expression (change >1.5 fold; P<0.05) between the PE and CTL cultures under 4% O_2_. A relatively small number of genes differed in expression between the PE and CTL cultures. Out of over 47,000 probes on the arrays, a total of 26 genes were up-regulated and 31 down-regulated in the PE versus CTL comparisons, although there was considerable heterogeneity across lines within the PE and CTL groups, and no gene on its own could unequivocally distinguish a PE from a CTL culture (Fig. 6A). Some lines, most notably D and possibly K, from the PE group appeared to display a transcriptome profile more resembling CTL than fellow PE lines. Even the twins (I & J) did not demonstrate identical profiles, although they were quite similar. A gene ontology (GO) analysis of the probes up-regulated in PE relative to CTL (Table S2A in File S2) indicated that the terms with the lowest P-values included a number of genes encoding proteins involved in chemokine and cytokine signaling (e.g. *CXCL1, CXCL6, IL8*), cell migration, extracellular matrix and cell adhesion processes (e.g. (*HAPLN1, PODXL,* and *STC1*), and metal binding (e.g. *MT1G, MT1A*). Many of those down-regulated in PE versus CTL have also been implicated in extracellular processes, including cell adhesion and matrix formation (Fig. 6B; Table S2B in File S2). The up-regulation of three genes, namely *HAPLN1* (P<0.002), *CXCL1*, (P<0.02), and possibly *IL8* (P<0.1), in PE versus CTL cells has been confirmed by real time quantitative PCR (Fig. S7 in File S1).

Both the CTL and PE lines showed shifts in the up-and down-regulation of many genes when cells were switched from 4% to 20% O_2_ culture conditions (Fig. 6C). A total of 497 were significantly up-regulated by shifting to 20% O_2_ (39 uniquely to CTL, 286 to PE, and 172 to both). Of those gene networks up-regulated in PE cells relative to CTL, the predominant terms affected related to stress reactions of the endoplasmic reticulum (unfolded protein response or UPR). Representative genes associated with the UPR, including *CALR, HERPUD1*, *VIMP, DERL2, HSPA5 (BIP)*, and *DDIT3 (CHOP)*, and *ATF4*, were up-regulated in PE cells relative to CTL by shifting from 4% to 20% O_2_ (Table S3 in File S2, Annotation Clusters 1 and 5; GO:0005783 and GO:0034976, respectively). Other up-regulated networks in PE included cell adhesion, and protein/RNA catabolism in the cytoplasm, all most probably indicative of cells under extreme stress (Table S3 in File S2, Annotation Clusters 2, 3 and 4). Genes up-regulated in response to 20% O_2_ and common to both PE and CTL again suggested dynamic changes in intracellular membranes of both types of cell and a strongly positive association with cell death (Table S4 in File S2, Annotation Clusters 2 and 3). There was also up-regulation of genes encoding transporters of amino acids, di-and tricarboxylic acids, and metal ions, consistent with increased aerobic metabolism (Table S4 in File S2, Annotation Clusters 3, 4 and 5). CTL, unlike PE cells, displayed a modest up-regulation in expression of gene networks associated with transcription and translation in response to 20% O_2_ relative to PE cells (Table S5 in File S2).

About 831 gene probes were down-regulated by changing culture conditions from 4% to 20% O_2_ (129 to CTL, 314 to PE and 388 to both) (Fig. 6C). The most significantly down-regulated components distinguishing PE cells from CTL under 20% O_2_ were ones involved in intracellular membrane processing, mitosis and the cell cycle, and the cytoskeleton (Table S6 in File S2). Gene networks equally affected in both the PE and CTL cells generally fell into these same categories (Table S7 in File S2). As anticipated, genes involved in carbohydrate metabolism and, in particular, glycolysis and the pentose phosphate pathway were strongly down-regulated in both PE and CTL cells cultured under 20% *versus* 4% O_2_. Genes more strongly down regulated in CTL than PE cells under 20% O_2_ conditions were associated with ribosome assembly (Table S8 in File S2, Annotation Clusters 1 and 2).

## Discussion

During a normal pregnancy, the development of the placenta through most of the first trimester occurs under oxygen tensions as low as 20 mm Hg (equivalent to ∼3% O_2_ gas atmosphere) [32], conditions that may promote the growth and invasiveness of EVT and limit its terminal differentiation [10], [33]. However, increased blood flow into the intervillous space follows breakdown of plugs of TB in the maternal spiral arteries and leads to enhanced perfusion of oxygenated blood through the placental bed after about the 10^th^ week of pregnancy [34], [35], which may slow growth and promote final maturation of EVT. When remodeling of the maternal spiral arteries is incomplete, the vessels are presumed to have retained much of their vasoreactivity, as is evident in term placentae from mothers with PE [36]. Under these circumstances, the overall flow of blood is probably lower than through the more flaccid, wider-bored vessels of a normal pregnancy but, at the same time, may be more forceful and irregular [10], [37]. Hence, the placental villi and syncytioTB, in particular, could be exposed to fluctuations in blood flow, oxygen availability and, most probably, ischemia-reperfusion injury [38], as well as metabolic stress resulting from hypoxia [8], [10], [39]. In view of the irregular perfusion of the placental bed in pregnancies complicated by PE, it is perhaps not surprising that placentae from children born to such women tend to exhibit greater biochemical signs of oxidative and nitrative stress than those who experienced normal term deliveries [10], [40]–[43]. In other words, the stressed condition of placentae from PE patients may be secondary to an erratic supply of O_2_. On the other hand, a second school of thought is that severe forms of PE arise from an inability of the TB to respond appropriately when oxygen tensions increase [44]–[46], as might occur when the EVT approach maternal blood vessels at the time the placenta is becoming established or when maternal blood perfusion of the mature placenta is erratic. To what extent non-TB tissues, such as fibroblasts from the UC, of PE infants, are also affected by changing availability of O_2_ has not been examined, but the data reported here suggest that there is measurable susceptibility compared to controls.

We chose to use UC to establish cultures for reprogramming to iPSC because such tissues provide a readily accessible source of primary cells representing the fetal-placental unit. It was a surprise that outgrowths were more difficult to establish from infants born to PE mothers than comparable tissues from CTL. This defect was especially noticeable in a sub-set of PE cords that completely failed to provide outgrowths under ambient oxygen conditions but did so readily at a much lower oxygen tension (Fig. 1A; Table 1). This sensitivity to ambient O_2_ and, to a lesser extent, to high glucose in the culture medium, was specific to PE. It was not evident in cultures derived from two UC (CTL #7 & 8) obtained after caesarian deliveries at approximately the same stage of pregnancy as some of the PE births, and so we suspect that gestation length and mode of delivery were not confounding factors. Gestational age also appeared not to be a factor in the greater sensitivity of PE cultures to a switch from 4% to 20% O_2_ and of PE cultures to tolerate DME (Fig. S6A & B in File S1). For similar reasons, it seems that treatment of the mothers with betamethasone, intended to accelerate fetal lung maturity, was also not a cause of the poorer ability of PE outgrowths to tolerate 20% O_2_. The culture derived from CTL (#7), an infant that had been exposed to betamethasone did not exhibit greater oxygen sensitivity than other CTL, while those from two PE infants (B & E) that had not been exposed to betamethasone behaved similarly to other PE lines (Fig. 1). Nonetheless, once established from the outgrowths, doubling times of PE cell lines that had been initiated under 20% O_2_ were no different than those of CTL lines initiated under the same conditions (Fig. 1C). However, all cell lines, whether PE or CTL, grew more slowly at 20% than they did at 4% O_2_ (Fig. 1C). These observations suggested to us that the PE and CTL cells differed in their initial response to ambient O_2_, but that, once adapted to those conditions, they behaved similarly.

The hypothesis that the PE and CTL cell lines might be distinguishable in response to an acute exposure to 20% O_2_ was strengthened in experiments where the cells were switched from 4% to higher 20% O_2_ conditions. After such a switch, the PE lines proliferated significantly more slowly than CTL (Fig. 2A) and acquired an elongated cell shape (Fig. 2B, Fig. S1 in File S1). This sensitivity of PE lines to oxidative stress was also evident when the fibroblast cultures were exposed to DEM under 4% O_2_ conditions (Fig. 4B; Fig. 5A & B). There was one additional striking difference between the two cell types, namely capacity of glutathione to rescue CTL but not PE cultures when exposed to DEM under 4% O_2_ conditions. We currently have no good explanation for this phenomenon, particularly as both PE and CTL cultures contained equivalent concentrations of reduced glutathione (Fig. 3).

One possible explanation for the differences between PE and CTL cells is that the cord-derived explants from PE pregnancies were so metabolically stressed at the time they were collected that they were poorly capable of tolerating sudden exposure to the non-physiological conditions imposed by 20% O_2_, while they adapted well to the more physiological 4% O_2_. In other words, PE fibroblasts may have a response system that poorly equips them to react to sudden changes in O_2_ availability. This is consistent with the proposal that severe PE is linked to defects in oxygen sensing and a failure to down-regulate expression of HIF proteins when O_2_ levels are high [44], [45], [47], [48], but there has been surprisingly little recent follow-up to these intriguing observations. Whatever the cause, the inability of UC fibroblasts to adjust to ambient O_2_ appears to be retained over multiple cell divisions at 4% O_2_, as the cells remain acutely sensitive when they are switched from low to the higher O_2_ conditions no matter what the passage (data not shown). The PE cells are either genetically distinct from the CTL or at some time in their history acquired a stable epigenetic memory that renders them less able than the CTL to endure a switch to high O_2_. It is important to recognize, however, that ambient O_2_ was damaging to the replicative capacity of all the cultures and not just to the more sensitive cells derived from PE pregnancies.

If the PE cells are either genetically or epigenetically distinct from CTL, we reasoned that these traits might be reflected in differences in gene expression and hence transcriptome profiles between the fibroblast cultures. There were a number of differences noted between PE and CTL cultures grown under 4% O_2_, but no one gene product was identified that could uniquely distinguish the two kinds of cells (Fig. 6), possibly reflecting the genetic complexity of the disease. An overall elevated expression of transcripts for IL8 and for other genes linked to cytokine signaling, metal binding, and products associated with the extracellular matrix and adhesion, could help explain the elevated concentrations of some of the corresponding protein products in blood of PE mothers and infants [49]–[53], but there was no obvious regulation of pathways anticipated to combat stress. In short, the microarrays comparing PE and CTL cells cultured under physiological O_2_ conditions revealed no immediate insight into the causes of PE, although several of the candidate genes identified as differentially expressed might be usefully explored in future studies.

A comparison of the gene expression profiles of PE and CTL switched to 20% O_2_ conditions revealed many more differences in gene expression (Fig. 6C), but, upon close examination, only appeared to reinforce the conclusion that the PE fibroblasts were undergoing a more severe unfolded protein response (UPR), dividing more slowly, and surviving less well than the CTL cells in response to the switch in O_2_ conditions. Clearly the 20% CTL cultures were on a downhill path too, but one less precipitous than that for PE. Therefore, the microarray data confirmed the increased vulnerability of PE fibroblast cultures to acute O_2_ toxicity but provided no clue as to what genes might play a causal role. Microarray studies performed on samples derived from placentae of PE and CTL patients have given a somewhat similar picture of increased metabolic stress, reduced proliferation and increased cell death in PE [54]–[59], but it is difficult to avoid the conclusion that these differences were a consequence of the contrasting conditions to which the placenta had been exposed in the weeks prior to delivery. Zhou et al. [60] cultured cytoTB isolated from term placentae and, by microarray noted some consistent differences in gene expression between such cells from PE placentae and those from CTL. However, these differences resolved over time in culture, suggesting that the phenomenon might be a reflection of the impaired past of the tissue rather than a basis of the disease. Even the intriguing observation that HIF proteins are not rapidly degraded in response to ambient O_2_ in TB of infants after severe PE [44]–[46] could be a consequence of past placental history.

Thus, while many studies have shown that placental cells from PE infants can be distinguished from CTL cells at birth of the infant, a link to the origins of the disease has not proven straightforward. Our chance observation that PE fibroblasts derived from UC demonstrate a consistent, intrinsic flaw in response to sudden exposure to high O_2_ and that this abnormality persists over multiple cell divisions may provide a useful clue to the basis of the early onset, usually severe, form of the disease. It will be of interest, therefore, to determine whether or not the O_2_ sensitivities of the PE fibroblast lines are retained when they are converted to iPSC and, subsequently, once they have been allowed to differentiate to trophoblast, but whether the basis of this abnormal response to O_2_ is genetic or epigenetic remains unclear. Our present hypothesis is that placental cells from conceptuses whose mothers develop PE early in their pregnancies are relatively intolerant of sudden changes to higher oxygen tensions as noted with the UC fibroblasts from PE cases in this study. As a result of this hypersensitivity, extravillous cytotrophoblast (EVT) cells are predicted to cease dividing and to differentiate and possibly die prematurely as they approach endometrial vessels carrying oxygenated blood, the consequence of which will be poor perfusion of the placenta. Similarly, we hypothesize that syncytiotrophoblast will only begin to encounter well-oxygenated maternal blood after the uteroplacental arteries “open” towards the end of the first trimester of pregnancy [15], [61] and that the oxygen hypersensitivity of these cells will cause them to turnover at an accelerated rate and shed cell contents and debris into the circulating maternal blood more quickly than normal syncytiotrophoblast. Thus, a combination of a shallow, poorly-perfused placenta and an unstable syncytiotrophoblast interface releasing abnormally high amounts of pro-inflammatory compounds capable of provoking inflammatory responses in the endothelium of susceptible mothers might lead to the clinical manifestations of maternal disease [9], [61].

## Supporting Information

File S1 Contains the following files: **Figure S1.** Cell morphologies of selected CTL and PE cell lines after switching them to 20% O_2_ at p 3 and culturing for a further 5 days (right nine panels). The cells in the nine left panels had been continuously maintained under 4% O_2_ for the same period in parallel. The cell lines shown are CTL lines (8, 9, 10), PE O_2_ sensitive (**O_2_-s**; G, I, J), and PE O_2_ non-sensitive (**O_2_-n**; D, L) with uncharacterized line A. Bar, 0.5 mm. **Figure S2.** Cell death associated with the presence of DEM in the culture medium. The figure shows the survival ratio (y-axis) of PE line N decreasing gradually with increasing concentrations of DEM (x-axis).Concentrations above 0.7 mM were sufficient to cause almost complete cell death within 24 h of exposure. **Figure S3.** Effect of tBHQ on growth of UC fibroblasts (A) Fibroblasts (four PE, L, M, N, O; four CTL, #6, 7, 8, 9) were treated with 0.16 mM tBHQ for 24 h and numbers of surviving cells assessed as in Fig. 4B. Three replicate measurements were made per cell line. Data were combined and shown as box and whisker plots. (B) Relative abilities of PE and CTL fibroblasts to survive 0.16 mM tBHQ. The data from Fig. S3A were combined for the four PE and four CTL lines (*P≤0.05). All lines had been maintained in 4% O_2_ conditions for five passages before exposure to tBHQ. **Figure S4.** The effects of increasing concentrations of DEM applied below the toxic dose and time of exposure on the proliferation of UC fibroblasts under 4% O_2_ conditions. Aliquots of 2×10^4^ cells of human umbilical cord fibroblast from CTL (#8) at p 5 were seeded into individual, gelatin coated wells of 6-well culture plates. They were cultured in 2 ml LG-medium in the presence of increasing concentrations of DEM (0 µM to 100 µM) for up to 96 h. Their growth was compared with that of the same cells grown without DEM but under 20% O_2_. The medium in each well was replaced once at 48 h. Cell numbers were counted from triplicate cultures at 24, 48, 72, and 96 h. Values are means ± SEM for triplicate cultures. At each time point, unattached, floating cells in the supernatant were collected and counted separately from the attached cells that had been released from the substratum with TrypLE. The percentages of floating (presumed dying or dead) cells relative to total cells were 0.8% (0 µM), 1.3% (40 µM), 1.6% (60 µM), 3.5% (80 µM) and 9.2% (100 µM), respectively, in the increasing concentrations of DEM under 4% O_2_. The percentage of floating cells in cultures maintained under 20% O_2_ without DEM was 3.8%. **Figure S5.** Cell cycle analyses of fibroblasts after 72 h of exposure to increasing concentrations of DEM. Aliquots of 2×10^4^ cells of UC fibroblast from CTL (#8) at p 5 were seeded into individual, gelatin coated wells of 6-well culture plates. They were cultured in 2 ml LG-medium in the presence of increasing concentrations of DEM (0 µM to 100 µM) for 72 h. **Figure S6.** (**A**) UC fibroblasts of four selected PE cases (C, D, F, M; mean of ages (weeks ± SEM) is 32.89±0.29, n = 4) and controls (#7 & 8; 33.50±0.50, n = 2) that were not different in gestational ages (P = 0.315) also showed similar differences of doubling time results when cultured under the four different conditions (HG/LG medium, high/low O_2_) as shown in Fig. 1C. The gray bars indicate PE lines and white bars indicate controls. (**B**) The selected fibroblasts also exhibited survival abilities consistent with Fig. 4B when 0.4 mM DEM was supplemented. The open triangle indicates M, one of the **O_2_**-s PE lines that were failed to form outgrowths in 20% O_2_ and closed triangles are other PE lines. Data analysis was by ANOVA (*P≤0.05; **P≤0.01; ***P≤0.001). **Figure S7.** Quantitative, real-time PCR analysis of four genes noted by microarray to be differentially up-regulated between PE and CTL fibroblasts that had been established and continuously cultured under 4% O_2_ conditions. Tested genes included: *HPLN1*, hyaluronan and proteoglycan link protein 1; *CXCL1*, chemokine (C-X-C motif) ligand 1 transcript variant 1; *IL8*, interleukin 8; *MT1G*, metallothionein 1G. RNA was extracted and analyzed from four PE lines (M, N, O, P) and three CTL lines (#7, 8, 9). The data are presented as fold-increase (± SEM) for PE versus CTL fibroblasts, (**P<0.01; *P<0.05). The values for *CXCL1* (P<0.1) would have been significant had one “outlier” been excluded. *MT1G* expression was very low in all samples as assessed with two sets of primers (Table S9 in File S2).
(DOCX)

File S2 Contains the following files: **Table S1.** List of human umbilical cord (UC) samples and their delivery conditions collected from 10 controls (CTL) and from 17 infants whose mothers suffered early onset preeclampsia (PE). **Table S2. A.** Gene ontology (GO) analysis of up-regulated genes in PE versus CTL UC fibroblasts in 4% O_2_ conditions. **B.** GO analysis of down-regulated genes in PE versus CTL UC fibroblasts in 4% O_2_ conditions. **Table S3.** Gene networks up-regulated in PE cells relative to CTL by shifting from 4% to 20% O_2_ culture conditions. **Table S4.** Gene networks up-regulated in both PE and CTL by shifting from 4% to 20% O_2_ culture conditions. **Table S5.** Gene networks up-regulated in CTL cells relative to PE by shifting from 4% to 20% O_2_ culture conditions. **Table S6.** Gene networks down-regulated in PE cells relative to CTL by shifting from 4% to 20% O_2_ culture conditions. **Table S7.** Gene networks down-regulated in both PE and CTL by shifting from 4% to 20% O_2_ culture conditions. **Table S8.** Gene networks down-regulated in CTL cells relative to PE by shifting from 4% to 20% O_2_ culture conditions. **Table S9.** Primers for coding sequence regions of four tested genes and the reference gene GAPDH.
(DOCX)
