# Supplementary material for: Abnormal Oxidative Stress Responses in Fibroblasts from Preeclampsia Infants
Source: PLoS One. 2014 Jul 24;9(7):e103110. doi: 10.1371/journal.pone.0103110 (PMC4110005; doi:10.1371/journal.pone.0103110)
Supplement: File S1 — Contains the following files: Figure S1. Cell morphologies of selected CTL and PE cell lines after switching them to 20% O2 at p 3 and culturing for a further 5 days (right nine panels). The cells in the nine left panels had been continuously maintained under 4% O2 for the same period in parallel. The cell lines shown are CTL lines (8, 9, 10), PE O2 sensitive (O2-s; G, I, J), and PE O2 non-sensitive (O2-n; D, L) with uncharacterized line A. Bar, 0.5 mm. Figure S2. Cell death associated with the presence of DEM in the culture medium. The figure shows the survival ratio (y-axis) of PE line N decreasing gradually with increasing concentrations of DEM (x-axis).Concentrations above 0.7 mM were sufficient to cause almost complete cell death within 24 h of exposure. Figure S3. Effect of tBHQ on growth of UC fibroblasts (A) Fibroblasts (four PE, L, M, N, O; four CTL, #6, 7, 8, 9) were treated with 0.16 mM tBHQ for 24 h and numbers of surviving cells assessed as in Fig. 4B. Three replicate measurements were made per cell line. Data were combined and shown as box and whisker plots. (B) Relative abilities of PE and CTL fibroblasts to survive 0.16 mM tBHQ. The data from Fig. S3A were combined for the four PE and four CTL lines (*P≤0.05). All lines had been maintained in 4% O2 conditions for five passages before exposure to tBHQ. Figure S4. The effects of increasing concentrations of DEM applied below the toxic dose and time of exposure on the proliferation of UC fibroblasts under 4% O2 conditions. Aliquots of 2×10^4^ cells of human umbilical cord fibroblast from CTL (#8) at p 5 were seeded into individual, gelatin coated wells of 6-well culture plates. They were cultured in 2 ml LG-medium in the presence of increasing concentrations of DEM (0 µM to 100 µM) for up to 96 h. Their growth was compared with that of the same cells grown without DEM but under 20% O2. The medium in each well was replaced once at 48 h. Cell numbers were counted from triplicate cultures at 24, 48, 72, and [file pone.0103110.s001.docx]

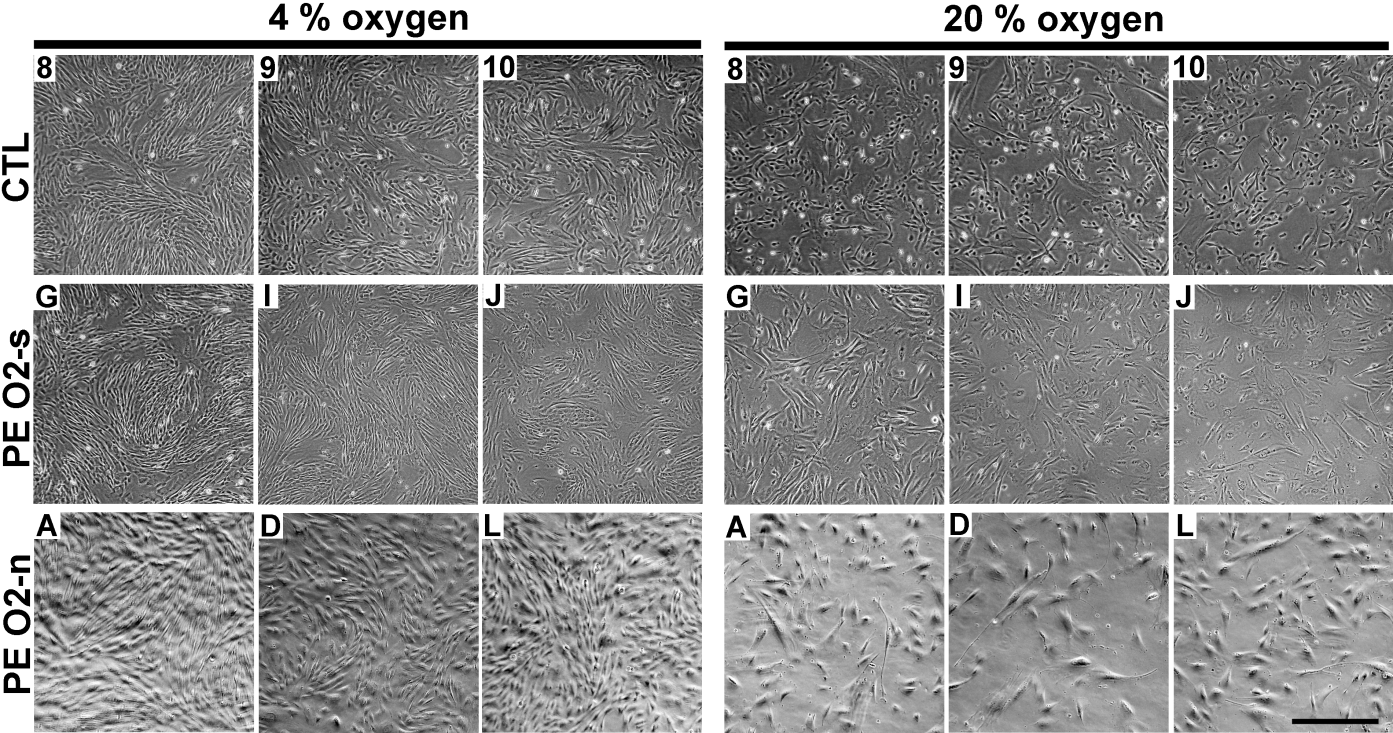


**Figure S1.**

Cell morphologies of selected CTL and PE cell lines after switching them to 20 % O_2_ at p 3 and culturing for a further 5 days (right nine panels). The cells in the nine left panels had been continuously maintained under 4 % O_2_ for the same period in parallel. The cell lines shown are CTL lines (8, 9, 10), PE O_2_ sensitive (**O2-s**; G, I, J), and PE O_2_ non-sensitive (**O2-n**; D, L) with uncharacterized line A. Bar, 0.5 mm.


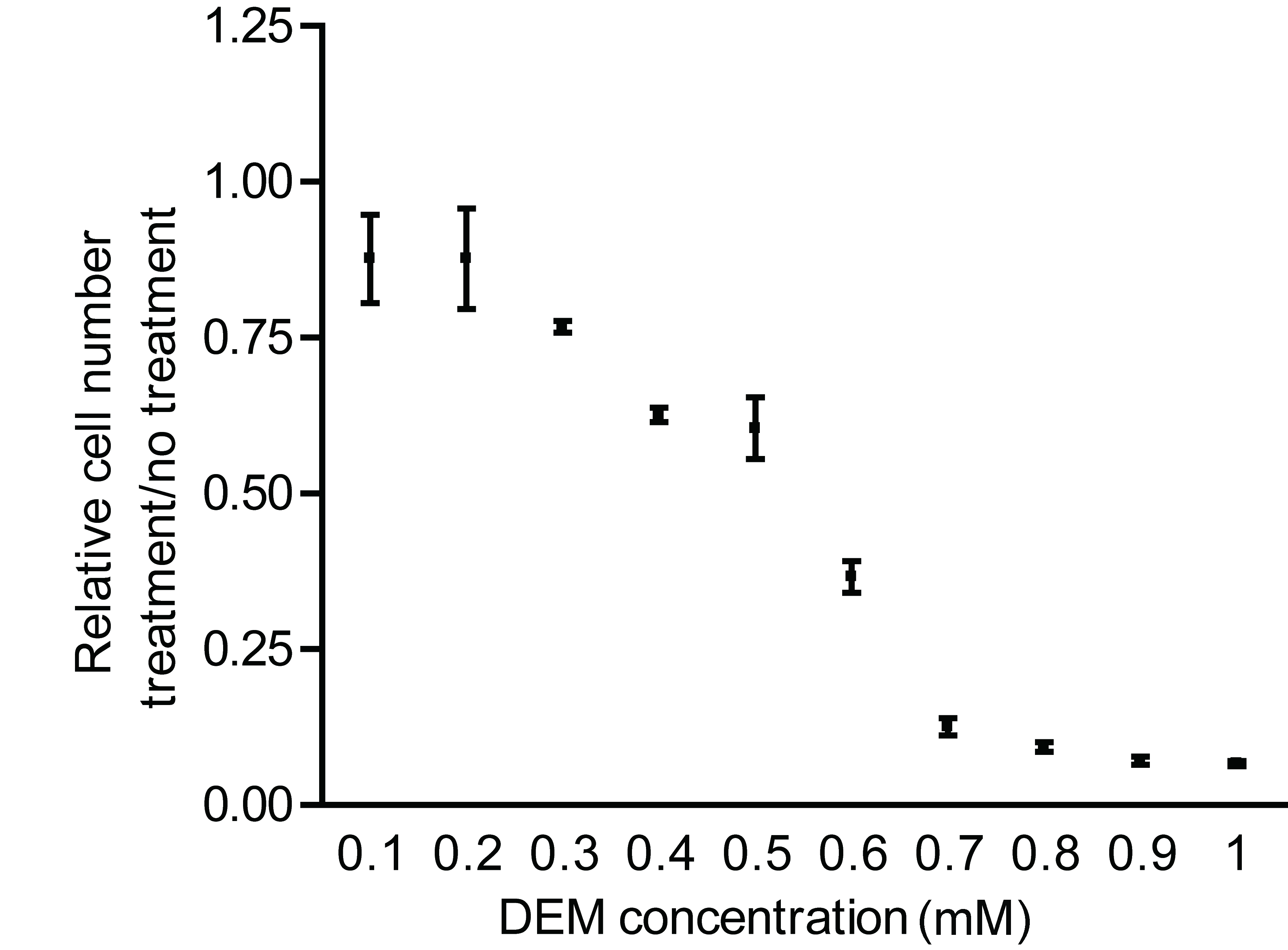


**Figure S2.**

Cell death associated with the presence of DEM in the culture medium. The figure shows the survival ratio (y-axis) of PE line N decreasing gradually with increasing concentrations of DEM (x-axis).Concentrations above 0.7 mM were sufficient to cause almost complete cell death within 24 h of exposure.





**Figure S3.**

**Effect of tBHQ on growth of UC fibroblasts** (A) Fibroblasts (four PE, L, M, N, O; four CTL, # 6, 7, 8, 9) were treated with 0.16 mM tBHQ for 24 h and numbers of surviving cells assessed as in Fig. 4B. Three replicate measurements were made per cell line. Data were combined and shown as box and whisker plots. (B) Relative abilities of PE and CTL fibroblasts to survive 0.16 mM tBHQ. The data from Fig. S3A were combined for the four PE and four CTL lines (* P ≤ 0.05). All lines had been maintained in 4 % O_2_ conditions for five passages before exposure to tBHQ.


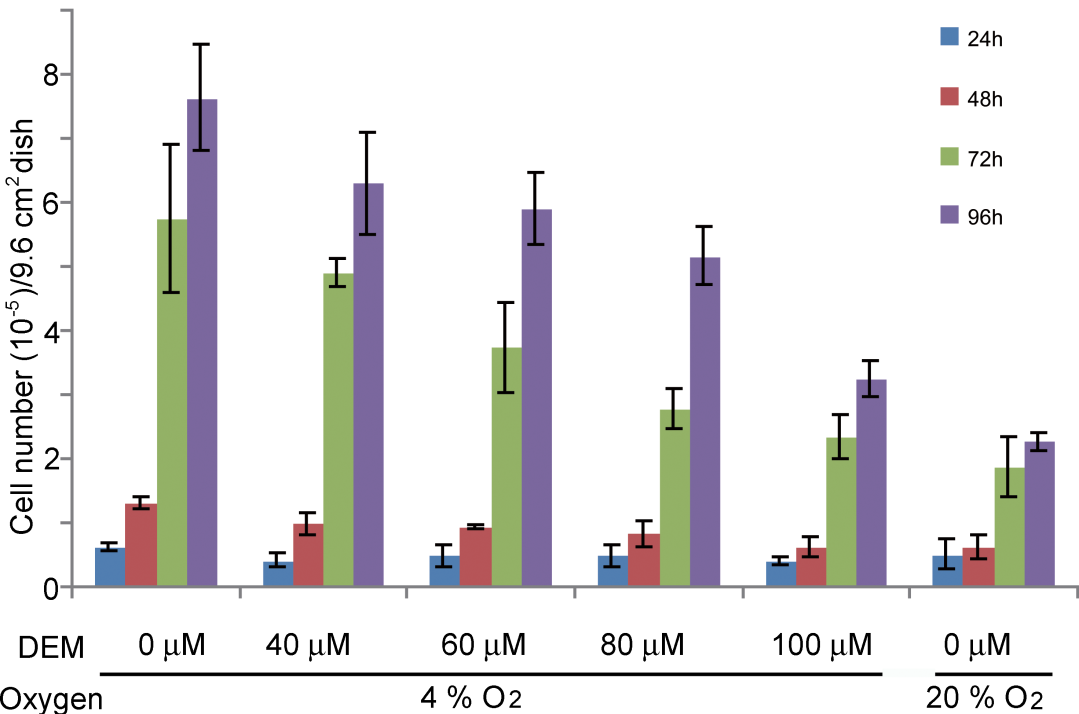


**Figure S4.**

The effects of increasing concentrations of DEM applied below the toxic dose and time of exposure on the proliferation of UC fibroblasts under 4 % O_2_ conditions. Aliquots of 2 x10^4^ cells of human umbilical cord fibroblast from CTL (# 8) at p 5 were seeded into individual, gelatin coated wells of 6-well culture plates. They were cultured in 2 ml LG-medium in the presence of increasing concentrations of DEM (0 µM to 100 µM) for up to 96 h. Their growth was compared with that of the same cells grown without DEM but under 20 % O_2_. The medium in each well was replaced once at 48 h. Cell numbers were counted from triplicate cultures at 24, 48, 72, and 96 h. Values are means ± SEM for triplicate cultures. At each time point, unattached, floating cells in the supernatant were collected and counted separately from the attached cells that had been released from the substratum with TrypLE. The percentages of floating (presumed dying or dead) cells relative to total cells were 0.8 % (0 μM), 1.3 % (40 μM), 1.6 % (60 μM), 3.5 % (80 μM) and 9.2 % (100 μM), respectively, in the increasing concentrations of DEM under 4 % O_2_. The percentage of floating cells in cultures maintained under 20 % O_2_ without DEM was 3.8 %.


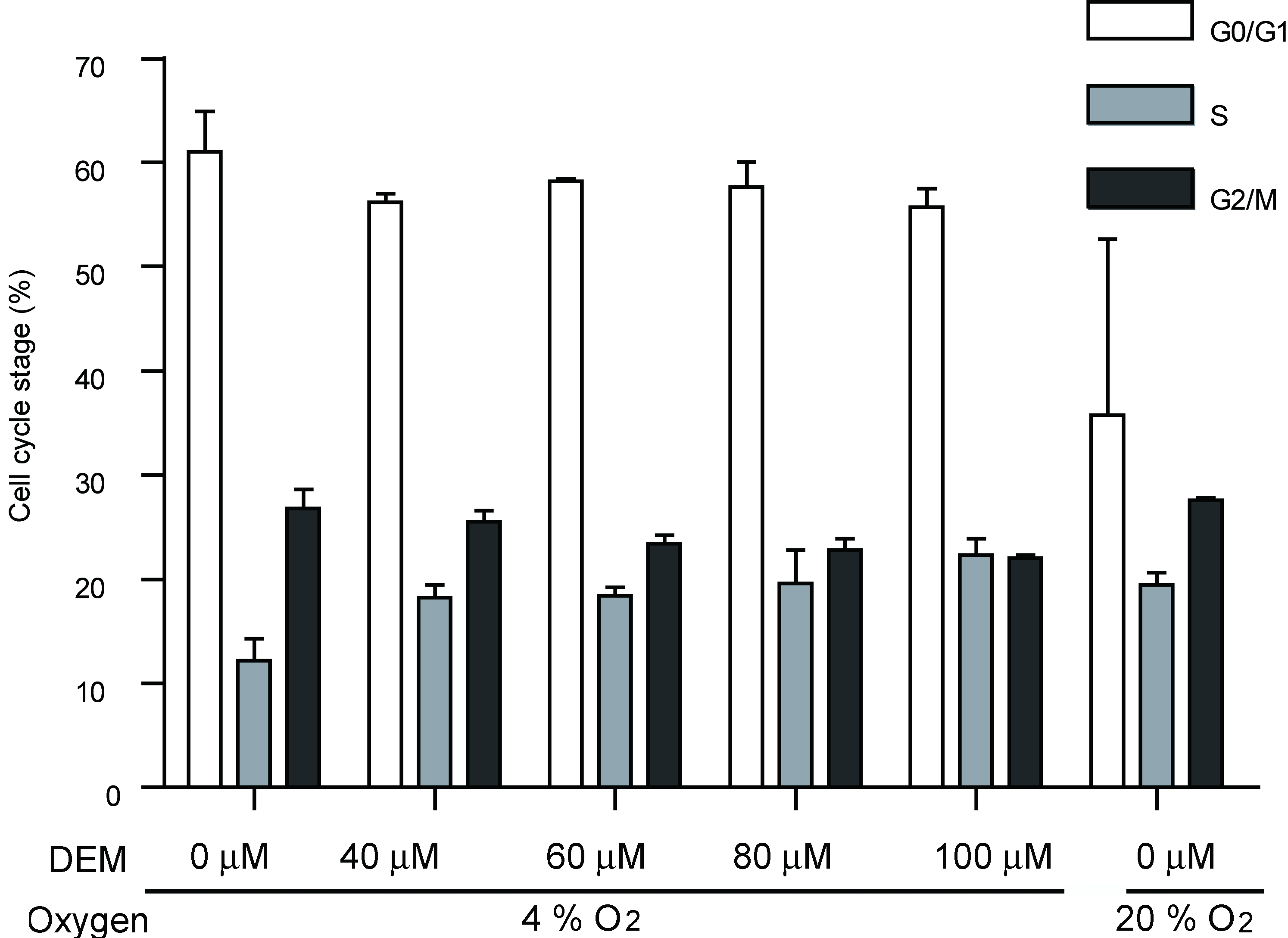


**Figure S5.**

Cell cycle analyses of fibroblasts after 72 h of exposure to increasing concentrations of DEM. Aliquots of 2x10^4^ cells of UC fibroblast from CTL (# 8) at p 5 were seeded into individual, gelatin coated wells of 6-well culture plates. They were cultured in 2 ml LG-medium in the presence of increasing concentrations of DEM (0 µM to 100 µM) for 72 h.


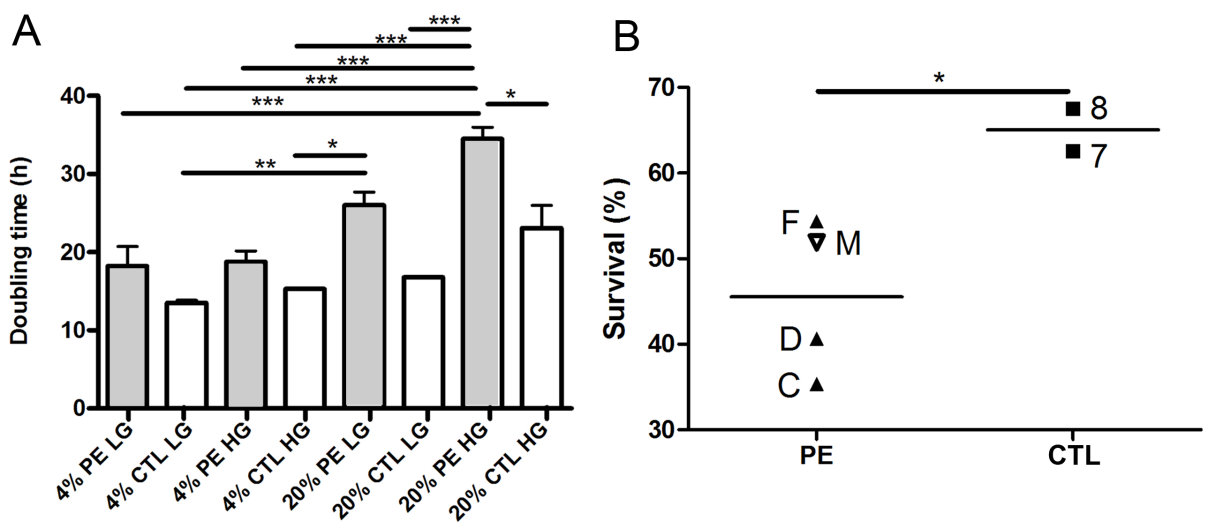


**Figure S6.**

(**A**) UC fibroblasts of four selected PE cases (C, D, F, M; mean of ages (weeks ± SEM) is 32.89 ± 0.29, n = 4) and controls (# 7 & 8; 33.50 ± 0.50, n = 2) that were not different in gestational ages (P = 0.315) also showed similar differences of doubling time results when cultured under the four different conditions (HG/LG medium, high/low O_2_) as shown in Fig. 1C. The gray bars indicate PE lines and white bars indicate controls.

(**B**) The selected fibroblasts also exhibited survival abilities consistent with Fig. 4B when 0.4 mM DEM was supplemented. The open triangle indicates M, one of the O2-s PE lines that were failed to form outgrowths in 20 % O_2_ and closed triangles are other PE lines. Data analysis was by ANOVA (* P ≤ 0.05; ** P ≤ 0.01; *** P ≤ 0.001).


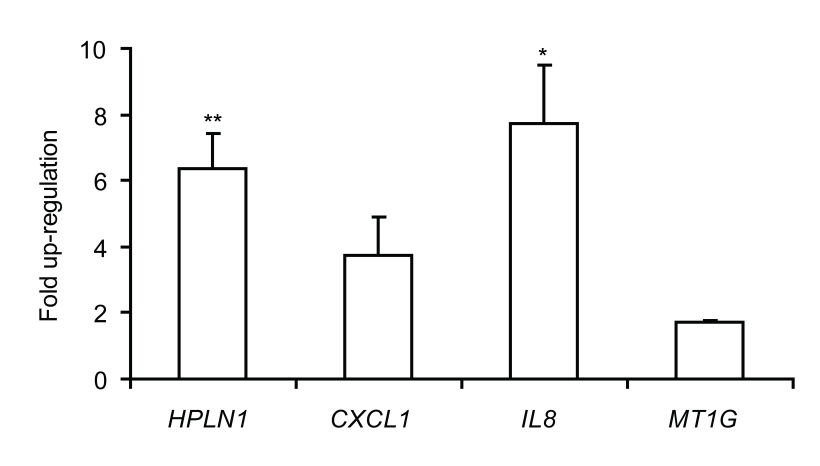


**Figure S7.**

Quantitative, real-time PCR analysis of four genes noted by microarray to be differentially up-regulated between PE and CTL fibroblasts that had been established and continuously cultured under 4 % O_2_ conditions. Tested genes included: *HPLN1*, hyaluronan and proteoglycan link protein 1; *CXCL1*, chemokine (C-X-C motif) ligand 1 transcript variant 1; *IL8*, interleukin 8; *MT1G*, metallothionein 1G. RNA was extracted and analyzed from four PE lines (M, N, O, P) and three CTL lines (# 7, 8, 9). The data are presented as fold-increase (± SEM) for PE versus CTL fibroblasts, (**P < 0.01; *P < 0.05). The values for *CXCL1* (P < 0.1) would have been significant had one “outlier” been excluded. *MT1G* expression was very low in all samples as assessed with two sets of primers (Table S9).
