# Supplementary material for: Abnormal Oxidative Stress Responses in Fibroblasts from Preeclampsia Infants
Source: PLoS One. 2014 Jul 24;9(7):e103110. doi: 10.1371/journal.pone.0103110 (PMC4110005; doi:10.1371/journal.pone.0103110)
Supplement: File S2 — Contains the following files: Table S1. List of human umbilical cord (UC) samples and their delivery conditions collected from 10 controls (CTL) and from 17 infants whose mothers suffered early onset preeclampsia (PE). Table S2. A. Gene ontology (GO) analysis of up-regulated genes in PE versus CTL UC fibroblasts in 4% O2 conditions. B. GO analysis of down-regulated genes in PE versus CTL UC fibroblasts in 4% O2 conditions. Table S3. Gene networks up-regulated in PE cells relative to CTL by shifting from 4% to 20% O2 culture conditions. Table S4. Gene networks up-regulated in both PE and CTL by shifting from 4% to 20% O2 culture conditions. Table S5. Gene networks up-regulated in CTL cells relative to PE by shifting from 4% to 20% O2 culture conditions. Table S6. Gene networks down-regulated in PE cells relative to CTL by shifting from 4% to 20% O2 culture conditions. Table S7. Gene networks down-regulated in both PE and CTL by shifting from 4% to 20% O2 culture conditions. Table S8. Gene networks down-regulated in CTL cells relative to PE by shifting from 4% to 20% O2 culture conditions. Table S9. Primers for coding sequence regions of four tested genes and the reference gene GAPDH. (DOCX) [file pone.0103110.s002.docx]

| Control  (CTL) Preeclampsia (PE) | Sample ID | sex | Gestational  age (week) | | Clinical  condition | Mode of delivery  CD = Cesarean del  SVD = spontaneous vaginal del | Betamethasone (B)  (x # of injections) | Microarray analysis of cells grown in 4%, 20% O_2_ |
| --- | --- | --- | --- | --- | --- | --- | --- | --- |
| CTL | 1 | M | 39 3/7 | Normal | | SVD | no | no no |
| CTL | 2 | F | 37 | Normal | | CD | no | yes yes |
| CTL | 3 | M | 39 2/7 | Normal | | SVD | no | yes yes |
| CTL | 4 | M | 40 | Normal | | CD (breech) | no | yes yes |
| CTL | 5 | F | 39 3/7 | Normal | | (Repeat) CD | no | yes yes |
| CTL | 6 | M | 40 | Normal | | SVD | no | yes yes |
| CTL | 7 | M | 34 | Preterm Premature Rupture of Membranes (PPROM) | | SVD | B (x 2) | yes yes |
| CTL | 8 | F | 33 | PPROM | | SVD | no | yes yes |
| CTL | 9 | F | 39 | Normal | | CD | no | yes yes |
| CTL | 10 | F | 39 1/7 | Normal | | CD | no | yes yes |
|  |  |  |  |  | |  |  |  |
| PE | A | F | 30 5/7 | HELLP syndrome | | CD | B (x 2) | yes yes |
| PE | B | M | 29 5/7 | severe PE | | CD | no, due to emergent delivery | yes yes |
| PE | C | M | 32 3/7 | severe PE | | CD | B (x 2) | yes yes |
| PE | D | M | 32 4/7 | severe PE | | CD | B (x 2) | yes yes |
| PE | E | M | 34 1/7 | HELLP syndrome | | CD | no, due to age >34 weeks | no no |
| PE | F | M | 32 6/7 | severe PE, IUGR | | CD | B (x 2) | yes yes |
| PE | G | M | 30 | severe PE on top of maternal hypertension | | (Repeat) CD | B (x 2) | yes yes |
| PE | H | M | 31 | severe PE | | (Repeat) CD | B (x 3) | yes yes |
| PE | I | M | 29 | severe PE, dichorionic/diammniotic twins | | (Repeat) CD | B (x 2) | yes yes |
| PE | J | M | 29 | severe PE, dichorionic/diammniotic twins | | (Repeat) CD | B (x 2) | yes yes |
| PE | K | M | 27 3/7 | severe PE | | CD | B (x 2) | yes yes |
| PE | L | M | 26 6/7 | severe PE | | CD (breech) | B (x 2) | no yes |
| PE | M | M | 33 5/7 | severe PE | | CD (breech) | B (x 2) | yes yes |
| PE | N | M | 28 5/7 | severe PE | | CD | B (x 2) | yes yes |
| PE | O | M | 28 5/7 | severe PE | | CD | B (x 2) | yes yes |
| PE | P | F | 31 5/7 | severe PE, IUGR | | CD | B (x 1) | yes yes |
| PE | Q | F | 27 | severe PE | | CD | B (x 2) | no no |

**Table S1.**

List of human umbilical cord samples and their delivery conditions collected from 10 controls (CTL) and from 17 infants whose mothers suffered early onset preeclampsia (PE).

| **Table S2A.**  GO analysis of up-regulated genes in PE versus CTL UC fibroblasts in 4% O_2_ conditions. | |
| --- | --- |
| Term | P-Value |
| chemokine activity | 0.0016 |
| chemokine receptor binding | 0.0018 |
| leukocyte migration | 0.002 |
| response to wounding | 0.0029 |
| extracellular region part | 0.0038 |
| cell migration | 0.0039 |
| cell motility | 0.0052 |
| localization of cell | 0.0052 |
| inflammatory response | 0.0061 |
| extracellular space | 0.0069 |
| carbohydrate binding | 0.01 |
| regulation of cell adhesion | 0.011 |
| cadmium ion binding | 0.013 |
| glycosaminoglycan binding | 0.014 |
| chemotaxis | 0.015 |
| taxis | 0.015 |
| cell surface receptor linked signal transduction | 0.016 |
| cell motion | 0.017 |
| pattern binding | 0.017 |
| polysaccharide binding | 0.017 |
| extracellular region | 0.021 |
| cytokine activity | 0.026 |
| GTPase activity | 0.03 |
| cell-cell signaling | 0.032 |
| defense response | 0.034 |
| microtubule-based process | 0.035 |
| microtubule | 0.04 |
| locomotory behavior | 0.041 |
| immune response | 0.045 |
| negative regulation of cell adhesion | 0.05 |
|  |  |
| **Table S2B.**  GO analysis of down-regulated genes in PE versus CTL UC fibroblasts in 4 % O_2_ conditions. | |
| Term | P-Value |
| extracellular region part | 1.50E-06 |
| extracellular region | 0.00018 |
| extracellular space | 0.00083 |
| cell adhesion | 0.0068 |
| biological adhesion | 0.0069 |
| extracellular matrix organization | 0.014 |
| calcium ion binding | 0.014 |
| proteinaceous extracellular matrix | 0.017 |
| extracellular matrix | 0.021 |
| extracellular structure organization | 0.033 |
| insulin-like growth factor binding | 0.04 |
| extracellular matrix binding | 0.043 |

**Table S3.**

Gene networks up-regulated in PE cells relative to CTL by shifting from 4 % to 20 % O_2_ culture conditions.

| Annotation Cluster 1 | Enrichment Score: 8.021957634798975 |  |  |  |
| --- | --- | --- | --- | --- |
| Category | Term | Count | % | P Value |
| GOTERM_CC_FAT | GO:0005783~endoplasmic reticulum | 50 | 19.92032 | 3.70E-15 |
| GOTERM_CC_FAT | GO:0044432~endoplasmic reticulum part | 29 | 11.55378 | 1.51E-13 |
| GOTERM_CC_FAT | GO:0042175~nuclear envelope-endoplasmic reticulum network | 20 | 7.968127 | 3.46E-08 |
| GOTERM_CC_FAT | GO:0005789~endoplasmic reticulum membrane | 19 | 7.569721 | 8.08E-08 |
| GOTERM_CC_FAT | GO:0012505~endomembrane system | 26 | 10.35857 | 1.84E-04 |
| GOTERM_CC_FAT | GO:0031090~organelle membrane | 29 | 11.55378 | 0.002565 |
| Annotation Cluster 2 | Enrichment Score: 4.261738457364617 |  |  |  |
| Category | Term | Count | % | P Value |
| GOTERM_BP_FAT | GO:0007155~cell adhesion | 27 | 10.75697 | 5.32E-06 |
| GOTERM_BP_FAT | GO:0022610~biological adhesion | 27 | 10.75697 | 5.46E-06 |
| GOTERM_BP_FAT | GO:0016337~cell-cell adhesion | 11 | 4.38247 | 0.005648 |
| Annotation Cluster 3 | Enrichment Score: 3.3478379426299956 |  |  |  |
| Category | Term | Count | % | P Value |
| GOTERM_CC_FAT | GO:0031988~membrane-bounded vesicle | 21 | 8.366534 | 2.46E-04 |
| GOTERM_CC_FAT | GO:0031982~vesicle | 23 | 9.163347 | 3.19E-04 |
| GOTERM_CC_FAT | GO:0016023~cytoplasmic membrane-bounded vesicle | 20 | 7.968127 | 4.48E-04 |
| GOTERM_CC_FAT | GO:0031410~cytoplasmic vesicle | 21 | 8.366534 | 0.001154 |
| Annotation Cluster 4 | Enrichment Score: 2.7657333531486405 |  |  |  |
| Category | Term | Count | % | P Value |
| GOTERM_CC_FAT | GO:0005773~vacuole | 13 | 5.179283 | 3.35E-04 |
| GOTERM_CC_FAT | GO:0005764~lysosome | 10 | 3.984064 | 0.003879 |
| GOTERM_CC_FAT | GO:0000323~lytic vacuole | 10 | 3.984064 | 0.003879 |
| Annotation Cluster 5 | Enrichment Score: 2.577483067977751 |  |  |  |
| Category | Term | Count | % | P Value |
| GOTERM_BP_FAT | GO:0034976~response to endoplasmic reticulum stress | 7 | 2.788845 | 6.88E-06 |
| GOTERM_BP_FAT | GO:0006984~ER-nuclear signaling pathway | 6 | 2.390438 | 1.20E-04 |
| GOTERM_BP_FAT | GO:0030968~endoplasmic reticulum unfolded protein response | 4 | 1.593625 | 0.00301 |
| GOTERM_BP_FAT | GO:0034620~cellular response to unfolded protein | 4 | 1.593625 | 0.00301 |
| GOTERM_BP_FAT | GO:0006983~ER overload response | 3 | 1.195219 | 0.008199 |
| GOTERM_BP_FAT | GO:0006986~response to unfolded protein | 4 | 1.593625 | 0.077989 |
| GOTERM_BP_FAT | GO:0051789~response to protein stimulus | 4 | 1.593625 | 0.190452 |
| Annotation Cluster 6 | Enrichment Score: 2.298373668138093 |  |  |  |
| Category | Term | Count | % | P Value |
| GOTERM_CC_FAT | GO:0044421~extracellular region part | 29 | 11.55378 | 3.38E-04 |
| GOTERM_CC_FAT | GO:0005578~proteinaceous extracellular matrix | 14 | 5.577689 | 8.60E-04 |
| GOTERM_CC_FAT | GO:0031012~extracellular matrix | 14 | 5.577689 | 0.001697 |
| GOTERM_CC_FAT | GO:0044420~extracellular matrix part | 7 | 2.788845 | 0.007413 |
| GOTERM_MF_FAT | GO:0005201~extracellular matrix structural constituent | 6 | 2.390438 | 0.008712 |
| GOTERM_CC_FAT | GO:0005615~extracellular space | 18 | 7.171315 | 0.023121 |
| GOTERM_CC_FAT | GO:0005576~extracellular region | 37 | 14.74104 | 0.110598 |
| Annotation Cluster 7 | Enrichment Score: 2.0745031405570864 |  |  |  |
| Category | Term | Count | % | P Value |
| GOTERM_BP_FAT | GO:0006928~cell motion | 16 | 6.374502 | 0.002899 |
| GOTERM_BP_FAT | GO:0016477~cell migration | 11 | 4.38247 | 0.005648 |
| GOTERM_BP_FAT | GO:0051674~localization of cell | 11 | 4.38247 | 0.011446 |
| GOTERM_BP_FAT | GO:0048870~cell motility | 11 | 4.38247 | 0.011446 |
| GOTERM_BP_FAT | GO:0001558~regulation of cell growth | 8 | 3.187251 | 0.019768 |
| Annotation Cluster 8 | Enrichment Score: 1.964746490775969 |  |  |  |
| Category | Term | Count | % | P Value |
| GOTERM_BP_FAT | GO:0043067~regulation of programmed cell death | 24 | 9.561753 | 0.001034 |
| GOTERM_BP_FAT | GO:0010941~regulation of cell death | 24 | 9.561753 | 0.001086 |
| GOTERM_BP_FAT | GO:0042981~regulation of apoptosis | 23 | 9.163347 | 0.002062 |
| GOTERM_BP_FAT | GO:0006916~anti-apoptosis | 8 | 3.187251 | 0.026755 |
| GOTERM_BP_FAT | GO:0043066~negative regulation of apoptosis | 10 | 3.984064 | 0.062441 |
| GOTERM_BP_FAT | GO:0043069~negative regulation of programmed cell death | 10 | 3.984064 | 0.06677 |
| GOTERM_BP_FAT | GO:0060548~negative regulation of cell death | 10 | 3.984064 | 0.068305 |
| Annotation Cluster 9 | Enrichment Score: 1.9272400150494635 |  |  |  |
| Category | Term | Count | % | P Value |
| GOTERM_BP_FAT | GO:0043062~extracellular structure organization | 9 | 3.585657 | 0.002131 |
| GOTERM_BP_FAT | GO:0030198~extracellular matrix organization | 7 | 2.788845 | 0.003449 |
| GOTERM_CC_FAT | GO:0044420~extracellular matrix part | 7 | 2.788845 | 0.007413 |
| GOTERM_MF_FAT | GO:0005201~extracellular matrix structural constituent | 6 | 2.390438 | 0.008712 |
| GOTERM_BP_FAT | GO:0030199~collagen fibril organization | 3 | 1.195219 | 0.062196 |
| GOTERM_CC_FAT | GO:0005581~collagen | 3 | 1.195219 | 0.092551 |
| Annotation Cluster 10 | Enrichment Score: 1.888525265225464 |  |  |  |
| Category | Term | Count | % | P Value |
| GOTERM_BP_FAT | GO:0006029~proteoglycan metabolic process | 6 | 2.390438 | 3.24E-04 |
| GOTERM_BP_FAT | GO:0006022~aminoglycan metabolic process | 5 | 1.992032 | 0.013115 |
| GOTERM_BP_FAT | GO:0030201~heparan sulfate proteoglycan metabolic process | 3 | 1.195219 | 0.015981 |
| GOTERM_BP_FAT | GO:0006790~sulfur metabolic process | 6 | 2.390438 | 0.023022 |
| GOTERM_BP_FAT | GO:0030203~glycosaminoglycan metabolic process | 4 | 1.593625 | 0.042004 |
| GOTERM_BP_FAT | GO:0005976~polysaccharide metabolic process | 5 | 1.992032 | 0.071115 |

**Table S4.**

Gene networks up-regulated in both PE and CTL by shifting from 4 % to 20 % O_2_ culture conditions.

| Annotation Cluster 1 | Enrichment Score: 4.389907843275318 |  |  |  |
| --- | --- | --- | --- | --- |
| Category | Term | Count | % | P Value |
| GOTERM_CC_FAT | GO:0005783~endoplasmic reticulum | 29 | 19.33333 | 3.19E-09 |
| GOTERM_CC_FAT | GO:0044432~endoplasmic reticulum part | 17 | 11.33333 | 2.84E-08 |
| GOTERM_CC_FAT | GO:0005789~endoplasmic reticulum membrane | 11 | 7.333333 | 8.42E-05 |
| GOTERM_CC_FAT | GO:0042175~nuclear envelope-endoplasmic reticulum network | 11 | 7.333333 | 1.32E-04 |
| GOTERM_CC_FAT | GO:0012505~endomembrane system | 12 | 8 | 0.06245 |
| GOTERM_CC_FAT | GO:0031090~organelle membrane | 15 | 10 | 0.07289 |
| Annotation Cluster 2 | Enrichment Score: 4.211515860473525 |  |  |  |
| Category | Term | Count | % | P Value |
| GOTERM_BP_FAT | GO:0042981~regulation of apoptosis | 22 | 14.66667 | 2.29E-06 |
| GOTERM_BP_FAT | GO:0043067~regulation of programmed cell death | 22 | 14.66667 | 2.68E-06 |
| GOTERM_BP_FAT | GO:0010941~regulation of cell death | 22 | 14.66667 | 2.84E-06 |
| GOTERM_BP_FAT | GO:0043065~positive regulation of apoptosis | 14 | 9.333333 | 5.49E-05 |
| GOTERM_BP_FAT | GO:0043068~positive regulation of programmed cell death | 14 | 9.333333 | 5.90E-05 |
| GOTERM_BP_FAT | GO:0010942~positive regulation of cell death | 14 | 9.333333 | 6.18E-05 |
| GOTERM_BP_FAT | GO:0006917~induction of apoptosis | 12 | 8 | 6.84E-05 |
| GOTERM_BP_FAT | GO:0012502~induction of programmed cell death | 12 | 8 | 7.03E-05 |
| GOTERM_BP_FAT | GO:0006915~apoptosis | 15 | 10 | 4.40E-04 |
| GOTERM_BP_FAT | GO:0012501~programmed cell death | 15 | 10 | 5.10E-04 |
| GOTERM_BP_FAT | GO:0008219~cell death | 16 | 10.66667 | 8.44E-04 |
| GOTERM_BP_FAT | GO:0016265~death | 16 | 10.66667 | 9.06E-04 |
| Annotation Cluster 3 | Enrichment Score: 3.0040492942710495 |  |  |  |
| Category | Term | Count | % | P Value |
| GOTERM_BP_FAT | GO:0042981~regulation of apoptosis | 22 | 14.66667 | 2.29E-06 |
| GOTERM_BP_FAT | GO:0043067~regulation of programmed cell death | 22 | 14.66667 | 2.68E-06 |
| GOTERM_BP_FAT | GO:0010941~regulation of cell death | 22 | 14.66667 | 2.84E-06 |
| GOTERM_BP_FAT | GO:0043066~negative regulation of apoptosis | 11 | 7.333333 | 7.04E-04 |
| GOTERM_BP_FAT | GO:0043069~negative regulation of programmed cell death | 11 | 7.333333 | 7.84E-04 |
| GOTERM_BP_FAT | GO:0060548~negative regulation of cell death | 11 | 7.333333 | 8.01E-04 |
| GOTERM_BP_FAT | GO:0008285~negative regulation of cell proliferation | 11 | 7.333333 | 8.18E-04 |
| GOTERM_BP_FAT | GO:0006916~anti-apoptosis | 6 | 4 | 0.028245 |
| GOTERM_BP_FAT | GO:0051050~positive regulation of transport | 6 | 4 | 0.037807 |
| GOTERM_BP_FAT | GO:0051240~positive regulation of multicellular organismal process | 4 | 2.666667 | 0.328809 |
| GOTERM_BP_FAT | GO:0051094~positive regulation of developmental process | 4 | 2.666667 | 0.405523 |
| Annotation Cluster 4 | Enrichment Score: 2.4946707393314735 |  |  |  |
| Category | Term | Count | % | P Value |
| GOTERM_BP_FAT | GO:0042592~homeostatic process | 18 | 12 | 1.42E-04 |
| GOTERM_BP_FAT | GO:0048878~chemical homeostasis | 14 | 9.333333 | 3.11E-04 |
| GOTERM_BP_FAT | GO:0055080~cation homeostasis | 10 | 6.666667 | 6.10E-04 |
| GOTERM_BP_FAT | GO:0055066~di-, tri-valent inorganic cation homeostasis | 9 | 6 | 8.28E-04 |
| GOTERM_BP_FAT | GO:0032469~endoplasmic reticulum calcium ion homeostasis | 3 | 2 | 0.001841 |
| GOTERM_BP_FAT | GO:0050801~ion homeostasis | 11 | 7.333333 | 0.002081 |
| GOTERM_BP_FAT | GO:0006874~cellular calcium ion homeostasis | 7 | 4.666667 | 0.004135 |
| GOTERM_BP_FAT | GO:0055074~calcium ion homeostasis | 7 | 4.666667 | 0.004714 |
| GOTERM_BP_FAT | GO:0006875~cellular metal ion homeostasis | 7 | 4.666667 | 0.005759 |
| GOTERM_BP_FAT | GO:0055065~metal ion homeostasis | 7 | 4.666667 | 0.007129 |
| GOTERM_BP_FAT | GO:0030005~cellular di-, tri-valent inorganic cation homeostasis | 7 | 4.666667 | 0.011448 |
| GOTERM_BP_FAT | GO:0006873~cellular ion homeostasis | 9 | 6 | 0.012445 |
| GOTERM_BP_FAT | GO:0055082~cellular chemical homeostasis | 9 | 6 | 0.013591 |
| GOTERM_BP_FAT | GO:0019725~cellular homeostasis | 10 | 6.666667 | 0.015203 |
| GOTERM_BP_FAT | GO:0030003~cellular cation homeostasis | 7 | 4.666667 | 0.018948 |
| Annotation Cluster 5 | Enrichment Score: 2.3428072653365097 |  |  |  |
| Category | Term | Count | % | P Value |
| GOTERM_MF_ALL | GO:0046943~carboxylic acid transmembrane transporter activity | 7 | 4.666667 | 7.05E-05 |
| GOTERM_MF_ALL | GO:0005342~organic acid transmembrane transporter activity | 7 | 4.666667 | 7.49E-05 |
| GOTERM_MF_ALL | GO:0015171~amino acid transmembrane transporter activity | 6 | 4 | 8.36E-05 |
| GOTERM_BP_FAT | GO:0046942~carboxylic acid transport | 8 | 5.333333 | 2.17E-04 |
| GOTERM_BP_FAT | GO:0015849~organic acid transport | 8 | 5.333333 | 2.26E-04 |
| GOTERM_MF_ALL | GO:0005275~amine transmembrane transporter activity | 6 | 4 | 2.46E-04 |
| GOTERM_MF_ALL | GO:0022804~active transmembrane transporter activity | 11 | 7.333333 | 4.43E-04 |
| GOTERM_MF_ALL | GO:0015175~neutral amino acid transmembrane transporter activity | 4 | 2.666667 | 5.14E-04 |
| GOTERM_BP_FAT | GO:0006865~amino acid transport | 6 | 4 | 8.93E-04 |
| GOTERM_BP_FAT | GO:0015837~amine transport | 6 | 4 | 0.002963 |
| GOTERM_MF_ALL | GO:0015300~solute:solute antiporter activity | 4 | 2.666667 | 0.007988 |
| GOTERM_BP_FAT | GO:0015804~neutral amino acid transport | 3 | 2 | 0.012874 |
| GOTERM_MF_ALL | GO:0015297~antiporter activity | 4 | 2.666667 | 0.014953 |
| GOTERM_MF_ALL | GO:0015179~L-amino acid transmembrane transporter activity | 3 | 2 | 0.03365 |
| GOTERM_MF_ALL | GO:0022857~transmembrane transporter activity | 13 | 8.666667 | 0.042241 |
| GOTERM_MF_ALL | GO:0022891~substrate-specific transmembrane transporter activity | 12 | 8 | 0.04955 |
| GOTERM_MF_ALL | GO:0022892~substrate-specific transporter activity | 13 | 8.666667 | 0.0628 |
| GOTERM_MF_ALL | GO:0015291~secondary active transmembrane transporter activity | 5 | 3.333333 | 0.072945 |
| GOTERM_MF_ALL | GO:0005215~transporter activity | 14 | 9.333333 | 0.109014 |
| GOTERM_MF_ALL | GO:0015075~ion transmembrane transporter activity | 7 | 4.666667 | 0.474508 |
| GOTERM_BP_FAT | GO:0006811~ion transport | 6 | 4 | 0.769309 |
| Annotation Cluster 6 | Enrichment Score: 2.1228114533607685 |  |  |  |
| Category | Term | Count | % | P Value |
| GOTERM_BP_FAT | GO:0008629~induction of apoptosis by intracellular signals | 5 | 3.333333 | 0.001024 |
| GOTERM_BP_FAT | GO:0009636~response to toxin | 5 | 3.333333 | 0.001616 |
| GOTERM_BP_FAT | GO:0048147~negative regulation of fibroblast proliferation | 3 | 2 | 0.001841 |
| GOTERM_BP_FAT | GO:0048145~regulation of fibroblast proliferation | 4 | 2.666667 | 0.002982 |
| GOTERM_BP_FAT | GO:0008630~DNA damage response, signal transduction resulting in induction of apoptosis | 3 | 2 | 0.030356 |
| GOTERM_BP_FAT | GO:0006974~response to DNA damage stimulus | 8 | 5.333333 | 0.035285 |
| GOTERM_BP_FAT | GO:0042770~DNA damage response, signal transduction | 3 | 2 | 0.141986 |
| Annotation Cluster 7 | Enrichment Score: 2.046005543062039 |  |  |  |
| Category | Term | Count | % | P Value |
| GOTERM_BP_FAT | GO:0031670~cellular response to nutrient | 3 | 2 | 0.001841 |
| GOTERM_BP_FAT | GO:0031668~cellular response to extracellular stimulus | 5 | 3.333333 | 0.001931 |
| GOTERM_BP_FAT | GO:0031669~cellular response to nutrient levels | 4 | 2.666667 | 0.005717 |
| GOTERM_BP_FAT | GO:0009991~response to extracellular stimulus | 7 | 4.666667 | 0.009913 |
| GOTERM_BP_FAT | GO:0031667~response to nutrient levels | 6 | 4 | 0.023879 |
| GOTERM_BP_FAT | GO:0007584~response to nutrient | 4 | 2.666667 | 0.110088 |
| Annotation Cluster 8 | Enrichment Score: 1.859894066540651 |  |  |  |
| Category | Term | Count | % | P Value |
| GOTERM_BP_FAT | GO:0002526~acute inflammatory response | 6 | 4 | 0.00131 |
| GOTERM_BP_FAT | GO:0009611~response to wounding | 13 | 8.666667 | 0.001445 |
| GOTERM_BP_FAT | GO:0006954~inflammatory response | 9 | 6 | 0.005571 |
| GOTERM_BP_FAT | GO:0006952~defense response | 9 | 6 | 0.137388 |
| GOTERM_BP_FAT | GO:0006955~immune response | 8 | 5.333333 | 0.346157 |
| Annotation Cluster 9 | Enrichment Score: 1.8539632244768693 |  |  |  |
| Category | Term | Count | % | P Value |
| GOTERM_CC_FAT | GO:0005773~vacuole | 8 | 5.333333 | 0.00522 |
| GOTERM_CC_FAT | GO:0000323~lytic vacuole | 7 | 4.666667 | 0.008565 |
| GOTERM_CC_FAT | GO:0005764~lysosome | 7 | 4.666667 | 0.008565 |
| GOTERM_CC_FAT | GO:0044437~vacuolar part | 3 | 2 | 0.100244 |
| Annotation Cluster 10 | Enrichment Score: 1.5966531780935416 |  |  |  |
| Category | Term | Count | % | P Value |
| GOTERM_CC_FAT | GO:0044421~extracellular region part | 16 | 10.66667 | 0.013703 |
| GOTERM_CC_FAT | GO:0005615~extracellular space | 12 | 8 | 0.028096 |
| GOTERM_CC_FAT | GO:0005576~extracellular region | 25 | 16.66667 | 0.042128 |

**Table S5.**

Gene networks up-regulated in CTL cells relative to PE by shifting from 4 % to 20 % O_2_ culture conditions.

| Annotation Cluster 1 | Enrichment Score: 4.389907843275318 |  |  |  |
| --- | --- | --- | --- | --- |
| Category | Term | Count | % | P Value |
| GOTERM_CC_FAT | GO:0005783~endoplasmic reticulum | 29 | 19.33333 | 3.19E-09 |
| GOTERM_CC_FAT | GO:0044432~endoplasmic reticulum part | 17 | 11.33333 | 2.84E-08 |
| GOTERM_CC_FAT | GO:0005789~endoplasmic reticulum membrane | 11 | 7.333333 | 8.42E-05 |
| GOTERM_CC_FAT | GO:0042175~nuclear envelope-endoplasmic reticulum network | 11 | 7.333333 | 1.32E-04 |
| GOTERM_CC_FAT | GO:0012505~endomembrane system | 12 | 8 | 0.06245 |
| GOTERM_CC_FAT | GO:0031090~organelle membrane | 15 | 10 | 0.07289 |
| Annotation Cluster 2 | Enrichment Score: 4.211515860473525 |  |  |  |
| Category | Term | Count | % | P Value |
| GOTERM_BP_FAT | GO:0042981~regulation of apoptosis | 22 | 14.66667 | 2.29E-06 |
| GOTERM_BP_FAT | GO:0043067~regulation of programmed cell death | 22 | 14.66667 | 2.68E-06 |
| GOTERM_BP_FAT | GO:0010941~regulation of cell death | 22 | 14.66667 | 2.84E-06 |
| GOTERM_BP_FAT | GO:0043065~positive regulation of apoptosis | 14 | 9.333333 | 5.49E-05 |
| GOTERM_BP_FAT | GO:0043068~positive regulation of programmed cell death | 14 | 9.333333 | 5.90E-05 |
| GOTERM_BP_FAT | GO:0010942~positive regulation of cell death | 14 | 9.333333 | 6.18E-05 |
| GOTERM_BP_FAT | GO:0006917~induction of apoptosis | 12 | 8 | 6.84E-05 |
| GOTERM_BP_FAT | GO:0012502~induction of programmed cell death | 12 | 8 | 7.03E-05 |
| GOTERM_BP_FAT | GO:0006915~apoptosis | 15 | 10 | 4.40E-04 |
| GOTERM_BP_FAT | GO:0012501~programmed cell death | 15 | 10 | 5.10E-04 |
| GOTERM_BP_FAT | GO:0008219~cell death | 16 | 10.66667 | 8.44E-04 |
| GOTERM_BP_FAT | GO:0016265~death | 16 | 10.66667 | 9.06E-04 |
| Annotation Cluster 3 | Enrichment Score: 3.0040492942710495 |  |  |  |
| Category | Term | Count | % | P Value |
| GOTERM_BP_FAT | GO:0042981~regulation of apoptosis | 22 | 14.66667 | 2.29E-06 |
| GOTERM_BP_FAT | GO:0043067~regulation of programmed cell death | 22 | 14.66667 | 2.68E-06 |
| GOTERM_BP_FAT | GO:0010941~regulation of cell death | 22 | 14.66667 | 2.84E-06 |
| GOTERM_BP_FAT | GO:0043066~negative regulation of apoptosis | 11 | 7.333333 | 7.04E-04 |
| GOTERM_BP_FAT | GO:0043069~negative regulation of programmed cell death | 11 | 7.333333 | 7.84E-04 |
| GOTERM_BP_FAT | GO:0060548~negative regulation of cell death | 11 | 7.333333 | 8.01E-04 |
| GOTERM_BP_FAT | GO:0008285~negative regulation of cell proliferation | 11 | 7.333333 | 8.18E-04 |
| GOTERM_BP_FAT | GO:0006916~anti-apoptosis | 6 | 4 | 0.028245 |
| GOTERM_BP_FAT | GO:0051050~positive regulation of transport | 6 | 4 | 0.037807 |
| GOTERM_BP_FAT | GO:0051240~positive regulation of multicellular organismal process | 4 | 2.666667 | 0.328809 |
| GOTERM_BP_FAT | GO:0051094~positive regulation of developmental process | 4 | 2.666667 | 0.405523 |
| Annotation Cluster 4 | Enrichment Score: 2.4946707393314735 |  |  |  |
| Category | Term | Count | % | P Value |
| GOTERM_BP_FAT | GO:0042592~homeostatic process | 18 | 12 | 1.42E-04 |
| GOTERM_BP_FAT | GO:0048878~chemical homeostasis | 14 | 9.333333 | 3.11E-04 |
| GOTERM_BP_FAT | GO:0055080~cation homeostasis | 10 | 6.666667 | 6.10E-04 |
| GOTERM_BP_FAT | GO:0055066~di-, tri-valent inorganic cation homeostasis | 9 | 6 | 8.28E-04 |
| GOTERM_BP_FAT | GO:0032469~endoplasmic reticulum calcium ion homeostasis | 3 | 2 | 0.001841 |
| GOTERM_BP_FAT | GO:0050801~ion homeostasis | 11 | 7.333333 | 0.002081 |
| GOTERM_BP_FAT | GO:0006874~cellular calcium ion homeostasis | 7 | 4.666667 | 0.004135 |
| GOTERM_BP_FAT | GO:0055074~calcium ion homeostasis | 7 | 4.666667 | 0.004714 |
| GOTERM_BP_FAT | GO:0006875~cellular metal ion homeostasis | 7 | 4.666667 | 0.005759 |
| GOTERM_BP_FAT | GO:0055065~metal ion homeostasis | 7 | 4.666667 | 0.007129 |
| GOTERM_BP_FAT | GO:0030005~cellular di-, tri-valent inorganic cation homeostasis | 7 | 4.666667 | 0.011448 |
| GOTERM_BP_FAT | GO:0006873~cellular ion homeostasis | 9 | 6 | 0.012445 |
| GOTERM_BP_FAT | GO:0055082~cellular chemical homeostasis | 9 | 6 | 0.013591 |
| GOTERM_BP_FAT | GO:0019725~cellular homeostasis | 10 | 6.666667 | 0.015203 |
| GOTERM_BP_FAT | GO:0030003~cellular cation homeostasis | 7 | 4.666667 | 0.018948 |
| Annotation Cluster 5 | Enrichment Score: 2.3428072653365097 |  |  |  |
| Category | Term | Count | % | P Value |
| GOTERM_MF_ALL | GO:0046943~carboxylic acid transmembrane transporter activity | 7 | 4.666667 | 7.05E-05 |
| GOTERM_MF_ALL | GO:0005342~organic acid transmembrane transporter activity | 7 | 4.666667 | 7.49E-05 |
| GOTERM_MF_ALL | GO:0015171~amino acid transmembrane transporter activity | 6 | 4 | 8.36E-05 |
| GOTERM_BP_FAT | GO:0046942~carboxylic acid transport | 8 | 5.333333 | 2.17E-04 |
| GOTERM_BP_FAT | GO:0015849~organic acid transport | 8 | 5.333333 | 2.26E-04 |
| GOTERM_MF_ALL | GO:0005275~amine transmembrane transporter activity | 6 | 4 | 2.46E-04 |
| GOTERM_MF_ALL | GO:0022804~active transmembrane transporter activity | 11 | 7.333333 | 4.43E-04 |
| GOTERM_MF_ALL | GO:0015175~neutral amino acid transmembrane transporter activity | 4 | 2.666667 | 5.14E-04 |
| GOTERM_BP_FAT | GO:0006865~amino acid transport | 6 | 4 | 8.93E-04 |
| GOTERM_BP_FAT | GO:0015837~amine transport | 6 | 4 | 0.002963 |
| GOTERM_MF_ALL | GO:0015300~solute:solute antiporter activity | 4 | 2.666667 | 0.007988 |
| GOTERM_BP_FAT | GO:0015804~neutral amino acid transport | 3 | 2 | 0.012874 |
| GOTERM_MF_ALL | GO:0015297~antiporter activity | 4 | 2.666667 | 0.014953 |
| GOTERM_MF_ALL | GO:0015179~L-amino acid transmembrane transporter activity | 3 | 2 | 0.03365 |
| GOTERM_MF_ALL | GO:0022857~transmembrane transporter activity | 13 | 8.666667 | 0.042241 |
| GOTERM_MF_ALL | GO:0022891~substrate-specific transmembrane transporter activity | 12 | 8 | 0.04955 |
| GOTERM_MF_ALL | GO:0022892~substrate-specific transporter activity | 13 | 8.666667 | 0.0628 |
| GOTERM_MF_ALL | GO:0015291~secondary active transmembrane transporter activity | 5 | 3.333333 | 0.072945 |
| GOTERM_MF_ALL | GO:0005215~transporter activity | 14 | 9.333333 | 0.109014 |
| GOTERM_MF_ALL | GO:0015075~ion transmembrane transporter activity | 7 | 4.666667 | 0.474508 |
| GOTERM_BP_FAT | GO:0006811~ion transport | 6 | 4 | 0.769309 |
| Annotation Cluster 6 | Enrichment Score: 2.1228114533607685 |  |  |  |
| Category | Term | Count | % | P Value |
| GOTERM_BP_FAT | GO:0008629~induction of apoptosis by intracellular signals | 5 | 3.333333 | 0.001024 |
| GOTERM_BP_FAT | GO:0009636~response to toxin | 5 | 3.333333 | 0.001616 |
| GOTERM_BP_FAT | GO:0048147~negative regulation of fibroblast proliferation | 3 | 2 | 0.001841 |
| GOTERM_BP_FAT | GO:0048145~regulation of fibroblast proliferation | 4 | 2.666667 | 0.002982 |
| GOTERM_BP_FAT | GO:0008630~DNA damage response, signal transduction resulting in induction of apoptosis | 3 | 2 | 0.030356 |
| GOTERM_BP_FAT | GO:0006974~response to DNA damage stimulus | 8 | 5.333333 | 0.035285 |
| GOTERM_BP_FAT | GO:0042770~DNA damage response, signal transduction | 3 | 2 | 0.141986 |
| Annotation Cluster 7 | Enrichment Score: 2.046005543062039 |  |  |  |
| Category | Term | Count | % | P Value |
| GOTERM_BP_FAT | GO:0031670~cellular response to nutrient | 3 | 2 | 0.001841 |
| GOTERM_BP_FAT | GO:0031668~cellular response to extracellular stimulus | 5 | 3.333333 | 0.001931 |
| GOTERM_BP_FAT | GO:0031669~cellular response to nutrient levels | 4 | 2.666667 | 0.005717 |
| GOTERM_BP_FAT | GO:0009991~response to extracellular stimulus | 7 | 4.666667 | 0.009913 |
| GOTERM_BP_FAT | GO:0031667~response to nutrient levels | 6 | 4 | 0.023879 |
| GOTERM_BP_FAT | GO:0007584~response to nutrient | 4 | 2.666667 | 0.110088 |
| Annotation Cluster 8 | Enrichment Score: 1.859894066540651 |  |  |  |
| Category | Term | Count | % | P Value |
| GOTERM_BP_FAT | GO:0002526~acute inflammatory response | 6 | 4 | 0.00131 |
| GOTERM_BP_FAT | GO:0009611~response to wounding | 13 | 8.666667 | 0.001445 |
| GOTERM_BP_FAT | GO:0006954~inflammatory response | 9 | 6 | 0.005571 |
| GOTERM_BP_FAT | GO:0006952~defense response | 9 | 6 | 0.137388 |
| GOTERM_BP_FAT | GO:0006955~immune response | 8 | 5.333333 | 0.346157 |
| Annotation Cluster 9 | Enrichment Score: 1.8539632244768693 |  |  |  |
| Category | Term | Count | % | P Value |
| GOTERM_CC_FAT | GO:0005773~vacuole | 8 | 5.333333 | 0.00522 |
| GOTERM_CC_FAT | GO:0000323~lytic vacuole | 7 | 4.666667 | 0.008565 |
| GOTERM_CC_FAT | GO:0005764~lysosome | 7 | 4.666667 | 0.008565 |
| GOTERM_CC_FAT | GO:0044437~vacuolar part | 3 | 2 | 0.100244 |
| Annotation Cluster 10 | Enrichment Score: 1.5966531780935416 |  |  |  |
| Category | Term | Count | % | P Value |
| GOTERM_CC_FAT | GO:0044421~extracellular region part | 16 | 10.66667 | 0.013703 |
| GOTERM_CC_FAT | GO:0005615~extracellular space | 12 | 8 | 0.028096 |
| GOTERM_CC_FAT | GO:0005576~extracellular region | 25 | 16.66667 | 0.042128 |

**Table S6.**

Gene networks down-regulated in PE cells relative to CTL by shifting from 4 % to 20 % O_2_ culture conditions.

| Annotation Cluster 1 | Enrichment Score: 11.998677481602153 |  |  |  |
| --- | --- | --- | --- | --- |
| Category | Term | Count | % | P Value |
| GOTERM_CC_FAT | GO:0043232~intracellular non-membrane-bounded organelle | 90 | 33.45725 | 9.04E-17 |
| GOTERM_CC_FAT | GO:0043228~non-membrane-bounded organelle | 90 | 33.45725 | 9.04E-17 |
| GOTERM_CC_FAT | GO:0005856~cytoskeleton | 39 | 14.49814 | 1.24E-04 |
| Annotation Cluster 2 | Enrichment Score: 11.859296589720744 |  |  |  |
| Category | Term | Count | % | P Value |
| GOTERM_CC_FAT | GO:0005694~chromosome | 36 | 13.3829 | 9.13E-16 |
| GOTERM_CC_FAT | GO:0044427~chromosomal part | 31 | 11.52416 | 7.77E-14 |
| GOTERM_CC_FAT | GO:0000775~chromosome, centromeric region | 14 | 5.204461 | 3.72E-08 |
| Annotation Cluster 3 | Enrichment Score: 8.213634117989013 |  |  |  |
| Category | Term | Count | % | P Value |
| GOTERM_BP_FAT | GO:0006259~DNA metabolic process | 30 | 11.15242 | 8.96E-10 |
| GOTERM_CC_FAT | GO:0005654~nucleoplasm | 38 | 14.12639 | 6.80E-09 |
| GOTERM_BP_FAT | GO:0006260~DNA replication | 17 | 6.319703 | 3.75E-08 |
| Annotation Cluster 4 | Enrichment Score: 8.174765989407257 |  |  |  |
| Category | Term | Count | % | P Value |
| GOTERM_CC_FAT | GO:0044427~chromosomal part | 31 | 11.52416 | 7.77E-14 |
| GOTERM_CC_FAT | GO:0000228~nuclear chromosome | 17 | 6.319703 | 2.27E-09 |
| GOTERM_CC_FAT | GO:0044454~nuclear chromosome part | 13 | 4.832714 | 2.56E-07 |
| GOTERM_CC_FAT | GO:0000785~chromatin | 13 | 4.832714 | 4.42E-05 |
| Annotation Cluster 5 | Enrichment Score: 7.502523452477168 |  |  |  |
| Category | Term | Count | % | P Value |
| GOTERM_CC_FAT | GO:0000793~condensed chromosome | 15 | 5.576208 | 6.97E-09 |
| GOTERM_CC_FAT | GO:0000776~kinetochore | 12 | 4.460967 | 1.67E-08 |
| GOTERM_CC_FAT | GO:0000775~chromosome, centromeric region | 14 | 5.204461 | 3.72E-08 |
| GOTERM_CC_FAT | GO:0000779~condensed chromosome, centromeric region | 11 | 4.089219 | 4.26E-08 |
| GOTERM_CC_FAT | GO:0000777~condensed chromosome kinetochore | 10 | 3.717472 | 1.67E-07 |
| Annotation Cluster 6 | Enrichment Score: 6.581444868094828 |  |  |  |
| Category | Term | Count | % | P Value |
| GOTERM_CC_FAT | GO:0005654~nucleoplasm | 38 | 14.12639 | 6.80E-09 |
| GOTERM_CC_FAT | GO:0031981~nuclear lumen | 48 | 17.84387 | 1.51E-07 |
| GOTERM_CC_FAT | GO:0070013~intracellular organelle lumen | 53 | 19.7026 | 7.27E-07 |
| GOTERM_CC_FAT | GO:0031974~membrane-enclosed lumen | 54 | 20.07435 | 1.13E-06 |
| GOTERM_CC_FAT | GO:0043233~organelle lumen | 53 | 19.7026 | 1.47E-06 |
| Annotation Cluster 7 | Enrichment Score: 5.110087090760189 |  |  |  |
| Category | Term | Count | % | P Value |
| GOTERM_BP_FAT | GO:0007017~microtubule-based process | 20 | 7.434944 | 1.25E-08 |
| GOTERM_BP_FAT | GO:0000226~microtubule cytoskeleton organization | 11 | 4.089219 | 9.06E-05 |
| GOTERM_BP_FAT | GO:0007010~cytoskeleton organization | 18 | 6.69145 | 4.14E-04 |
| Annotation Cluster 8 | Enrichment Score: 5.083667221230661 |  |  |  |
| Category | Term | Count | % | P Value |
| GOTERM_BP_FAT | GO:0051258~protein polymerization | 11 | 4.089219 | 2.90E-09 |
| GOTERM_BP_FAT | GO:0034621~cellular macromolecular complex subunit organization | 24 | 8.921933 | 6.51E-09 |
| GOTERM_BP_FAT | GO:0007017~microtubule-based process | 20 | 7.434944 | 1.25E-08 |
| GOTERM_BP_FAT | GO:0034622~cellular macromolecular complex assembly | 22 | 8.178439 | 1.99E-08 |
| GOTERM_BP_FAT | GO:0043623~cellular protein complex assembly | 16 | 5.947955 | 2.86E-08 |
| GOTERM_BP_FAT | GO:0043933~macromolecular complex subunit organization | 32 | 11.89591 | 1.34E-07 |
| GOTERM_BP_FAT | GO:0065003~macromolecular complex assembly | 30 | 11.15242 | 3.65E-07 |
| GOTERM_CC_FAT | GO:0015630~microtubule cytoskeleton | 25 | 9.29368 | 2.06E-06 |
| GOTERM_BP_FAT | GO:0006461~protein complex assembly | 24 | 8.921933 | 3.19E-06 |
| GOTERM_BP_FAT | GO:0070271~protein complex biogenesis | 24 | 8.921933 | 3.19E-06 |
| GOTERM_CC_FAT | GO:0005874~microtubule | 17 | 6.319703 | 3.28E-06 |
| GOTERM_CC_FAT | GO:0005856~cytoskeleton | 39 | 14.49814 | 1.24E-04 |
| GOTERM_BP_FAT | GO:0007018~microtubule-based movement | 9 | 3.345725 | 3.51E-04 |
| GOTERM_CC_FAT | GO:0044430~cytoskeletal part | 29 | 10.78067 | 3.85E-04 |
| GOTERM_MF_FAT | GO:0003924~GTPase activity | 12 | 4.460967 | 4.47E-04 |
| GOTERM_MF_FAT | GO:0005198~structural molecule activity | 21 | 7.806691 | 0.001983 |
| GOTERM_MF_FAT | GO:0005525~GTP binding | 12 | 4.460967 | 0.030532 |
| GOTERM_MF_FAT | GO:0032561~guanyl ribonucleotide binding | 12 | 4.460967 | 0.035626 |
| GOTERM_MF_FAT | GO:0019001~guanyl nucleotide binding | 12 | 4.460967 | 0.035626 |
| Annotation Cluster 9 | Enrichment Score: 5.0645842232838705 |  |  |  |
| Category | Term | Count | % | P Value |
| GOTERM_BP_FAT | GO:0007049~cell cycle | 40 | 14.86989 | 4.00E-11 |
| GOTERM_BP_FAT | GO:0022402~cell cycle process | 29 | 10.78067 | 4.26E-08 |
| GOTERM_BP_FAT | GO:0000279~M phase | 19 | 7.063197 | 3.29E-06 |
| GOTERM_BP_FAT | GO:0000278~mitotic cell cycle | 20 | 7.434944 | 4.40E-06 |
| GOTERM_BP_FAT | GO:0022403~cell cycle phase | 21 | 7.806691 | 6.08E-06 |
| GOTERM_BP_FAT | GO:0007067~mitosis | 13 | 4.832714 | 1.53E-04 |
| GOTERM_BP_FAT | GO:0000280~nuclear division | 13 | 4.832714 | 1.53E-04 |
| GOTERM_BP_FAT | GO:0000087~M phase of mitotic cell cycle | 13 | 4.832714 | 1.82E-04 |
| GOTERM_BP_FAT | GO:0048285~organelle fission | 13 | 4.832714 | 2.23E-04 |
| GOTERM_BP_FAT | GO:0051301~cell division | 11 | 4.089219 | 0.015955 |
| Annotation Cluster 10 | Enrichment Score: 4.82724747165611 |  |  |  |
| Category | Term | Count | % | P Value |
| GOTERM_BP_FAT | GO:0006259~DNA metabolic process | 30 | 11.15242 | 8.96E-10 |
| GOTERM_BP_FAT | GO:0033554~cellular response to stress | 22 | 8.178439 | 1.72E-04 |
| GOTERM_BP_FAT | GO:0006974~response to DNA damage stimulus | 17 | 6.319703 | 2.13E-04 |
| GOTERM_BP_FAT | GO:0006281~DNA repair | 13 | 4.832714 | 0.001498 |

**Table S7.**

Gene networks down-regulated in both PE and CTL by shifting from 4 % to 20 % O_2_ culture conditions.

| Annotation Cluster 1 | Enrichment Score: 31.07596403672504 |  |  |  |
| --- | --- | --- | --- | --- |
| Category | Term | Count | % | P Value |
| GOTERM_BP_FAT | GO:0000279~M phase | 61 | 18.82716 | 9.49E-43 |
| GOTERM_BP_FAT | GO:0007067~mitosis | 50 | 15.4321 | 3.46E-39 |
| GOTERM_BP_FAT | GO:0000280~nuclear division | 50 | 15.4321 | 3.46E-39 |
| GOTERM_BP_FAT | GO:0000087~M phase of mitotic cell cycle | 50 | 15.4321 | 8.81E-39 |
| GOTERM_BP_FAT | GO:0048285~organelle fission | 50 | 15.4321 | 2.75E-38 |
| GOTERM_BP_FAT | GO:0022403~cell cycle phase | 62 | 19.1358 | 7.89E-38 |
| GOTERM_BP_FAT | GO:0000278~mitotic cell cycle | 58 | 17.90123 | 2.32E-36 |
| GOTERM_BP_FAT | GO:0007049~cell cycle | 77 | 23.76543 | 7.18E-35 |
| GOTERM_BP_FAT | GO:0022402~cell cycle process | 65 | 20.06173 | 9.00E-33 |
| GOTERM_BP_FAT | GO:0051301~cell division | 45 | 13.88889 | 2.14E-27 |
| GOTERM_CC_FAT | GO:0043228~non-membrane-bounded organelle | 115 | 35.49383 | 2.20E-26 |
| GOTERM_CC_FAT | GO:0043232~intracellular non-membrane-bounded organelle | 115 | 35.49383 | 2.20E-26 |
| GOTERM_CC_FAT | GO:0015630~microtubule cytoskeleton | 54 | 16.66667 | 6.43E-26 |
| GOTERM_CC_FAT | GO:0005819~spindle | 31 | 9.567901 | 2.87E-24 |
| GOTERM_CC_FAT | GO:0044430~cytoskeletal part | 61 | 18.82716 | 8.26E-20 |
| GOTERM_CC_FAT | GO:0005856~cytoskeleton | 72 | 22.22222 | 1.19E-18 |
| Annotation Cluster 2 | Enrichment Score: 14.170424817492016 |  |  |  |
| Category | Term | Count | % | P Value |
| GOTERM_CC_FAT | GO:0005694~chromosome | 44 | 13.58025 | 1.91E-20 |
| GOTERM_CC_FAT | GO:0000793~condensed chromosome | 24 | 7.407407 | 1.94E-17 |
| GOTERM_CC_FAT | GO:0044427~chromosomal part | 37 | 11.41975 | 3.72E-17 |
| GOTERM_CC_FAT | GO:0000775~chromosome, centromeric region | 22 | 6.790123 | 1.62E-15 |
| GOTERM_CC_FAT | GO:0000776~kinetochore | 16 | 4.938272 | 2.41E-12 |
| GOTERM_CC_FAT | GO:0000779~condensed chromosome, centromeric region | 14 | 4.320988 | 6.29E-11 |
| GOTERM_CC_FAT | GO:0000777~condensed chromosome kinetochore | 13 | 4.012346 | 1.90E-10 |
| Annotation Cluster 3 | Enrichment Score: 13.829055672819774 |  |  |  |
| Category | Term | Count | % | P Value |
| GOTERM_BP_FAT | GO:0007017~microtubule-based process | 35 | 10.80247 | 8.16E-20 |
| GOTERM_BP_FAT | GO:0000226~microtubule cytoskeleton organization | 22 | 6.790123 | 3.92E-13 |
| GOTERM_BP_FAT | GO:0007051~spindle organization | 14 | 4.320988 | 1.08E-12 |
| GOTERM_BP_FAT | GO:0007010~cytoskeleton organization | 35 | 10.80247 | 1.40E-12 |
| Annotation Cluster 4 | Enrichment Score: 8.072482541258372 |  |  |  |
| Category | Term | Count | % | P Value |
| GOTERM_BP_FAT | GO:0007059~chromosome segregation | 19 | 5.864198 | 6.88E-15 |
| GOTERM_BP_FAT | GO:0000070~mitotic sister chromatid segregation | 13 | 4.012346 | 1.19E-12 |
| GOTERM_BP_FAT | GO:0000819~sister chromatid segregation | 13 | 4.012346 | 1.73E-12 |
| GOTERM_BP_FAT | GO:0051276~chromosome organization | 31 | 9.567901 | 8.34E-09 |
| GOTERM_BP_FAT | GO:0007076~mitotic chromosome condensation | 5 | 1.54321 | 7.48E-05 |
| GOTERM_BP_FAT | GO:0030261~chromosome condensation | 6 | 1.851852 | 8.60E-05 |
| GOTERM_CC_FAT | GO:0000796~condensin complex | 3 | 0.925926 | 0.004114 |
| Annotation Cluster 5 | Enrichment Score: 7.238268129160342 |  |  |  |
| Category | Term | Count | % | P Value |
| GOTERM_CC_FAT | GO:0005874~microtubule | 25 | 7.716049 | 4.15E-11 |
| GOTERM_BP_FAT | GO:0007018~microtubule-based movement | 17 | 5.246914 | 3.07E-10 |
| GOTERM_MF_FAT | GO:0003777~microtubule motor activity | 10 | 3.08642 | 5.81E-06 |
| GOTERM_MF_FAT | GO:0003774~motor activity | 11 | 3.395062 | 1.51E-04 |
| Annotation Cluster 6 | Enrichment Score: 6.016586315672334 |  |  |  |
| Category | Term | Count | % | P Value |
| GOTERM_BP_FAT | GO:0006096~glycolysis | 12 | 3.703704 | 7.57E-10 |
| GOTERM_BP_FAT | GO:0006007~glucose catabolic process | 12 | 3.703704 | 8.26E-09 |
| GOTERM_BP_FAT | GO:0019320~hexose catabolic process | 12 | 3.703704 | 5.52E-08 |
| GOTERM_BP_FAT | GO:0046365~monosaccharide catabolic process | 12 | 3.703704 | 7.49E-08 |
| GOTERM_BP_FAT | GO:0046164~alcohol catabolic process | 12 | 3.703704 | 3.01E-07 |
| GOTERM_BP_FAT | GO:0044275~cellular carbohydrate catabolic process | 12 | 3.703704 | 4.96E-07 |
| GOTERM_BP_FAT | GO:0016052~carbohydrate catabolic process | 12 | 3.703704 | 6.00E-06 |
| GOTERM_BP_FAT | GO:0019318~hexose metabolic process | 15 | 4.62963 | 1.51E-05 |
| GOTERM_BP_FAT | GO:0006006~glucose metabolic process | 13 | 4.012346 | 3.02E-05 |
| GOTERM_BP_FAT | GO:0005996~monosaccharide metabolic process | 15 | 4.62963 | 7.52E-05 |
| GOTERM_BP_FAT | GO:0006091~generation of precursor metabolites and energy | 16 | 4.938272 | 8.25E-04 |
| Annotation Cluster 7 | Enrichment Score: 5.43029207675065 |  |  |  |
| Category | Term | Count | % | P Value |
| GOTERM_BP_FAT | GO:0051726~regulation of cell cycle | 31 | 9.567901 | 7.14E-13 |
| GOTERM_BP_FAT | GO:0007346~regulation of mitotic cell cycle | 19 | 5.864198 | 4.72E-10 |
| GOTERM_BP_FAT | GO:0007093~mitotic cell cycle checkpoint | 10 | 3.08642 | 7.86E-08 |
| GOTERM_BP_FAT | GO:0000075~cell cycle checkpoint | 13 | 4.012346 | 1.20E-07 |
| GOTERM_BP_FAT | GO:0010564~regulation of cell cycle process | 14 | 4.320988 | 2.04E-07 |
| GOTERM_CC_FAT | GO:0000922~spindle pole | 8 | 2.469136 | 1.34E-06 |
| GOTERM_BP_FAT | GO:0031577~spindle checkpoint | 6 | 1.851852 | 1.56E-06 |
| GOTERM_BP_FAT | GO:0033043~regulation of organelle organization | 17 | 5.246914 | 3.19E-06 |
| GOTERM_BP_FAT | GO:0007088~regulation of mitosis | 9 | 2.777778 | 8.76E-06 |
| GOTERM_BP_FAT | GO:0051783~regulation of nuclear division | 9 | 2.777778 | 8.76E-06 |
| GOTERM_BP_FAT | GO:0045841~negative regulation of mitotic metaphase/anaphase transition | 5 | 1.54321 | 3.56E-05 |
| GOTERM_BP_FAT | GO:0007094~mitotic cell cycle spindle assembly checkpoint | 5 | 1.54321 | 3.56E-05 |
| GOTERM_BP_FAT | GO:0030071~regulation of mitotic metaphase/anaphase transition | 6 | 1.851852 | 4.46E-05 |
| GOTERM_BP_FAT | GO:0045839~negative regulation of mitosis | 5 | 1.54321 | 5.25E-05 |
| GOTERM_BP_FAT | GO:0051784~negative regulation of nuclear division | 5 | 1.54321 | 5.25E-05 |
| GOTERM_BP_FAT | GO:0051129~negative regulation of cellular component organization | 11 | 3.395062 | 3.35E-04 |
| GOTERM_BP_FAT | GO:0010639~negative regulation of organelle organization | 8 | 2.469136 | 8.49E-04 |
| GOTERM_BP_FAT | GO:0010948~negative regulation of cell cycle process | 5 | 1.54321 | 0.001109 |
| GOTERM_BP_FAT | GO:0045786~negative regulation of cell cycle | 7 | 2.160494 | 0.004062 |
| Annotation Cluster 8 | Enrichment Score: 4.809359262237588 |  |  |  |
| Category | Term | Count | % | P Value |
| GOTERM_MF_FAT | GO:0032553~ribonucleotide binding | 57 | 17.59259 | 5.53E-06 |
| GOTERM_MF_FAT | GO:0032555~purine ribonucleotide binding | 57 | 17.59259 | 5.53E-06 |
| GOTERM_MF_FAT | GO:0017076~purine nucleotide binding | 58 | 17.90123 | 9.85E-06 |
| GOTERM_MF_FAT | GO:0001882~nucleoside binding | 51 | 15.74074 | 1.31E-05 |
| GOTERM_MF_FAT | GO:0005524~ATP binding | 48 | 14.81481 | 1.31E-05 |
| GOTERM_MF_FAT | GO:0032559~adenyl ribonucleotide binding | 48 | 14.81481 | 1.86E-05 |
| GOTERM_MF_FAT | GO:0001883~purine nucleoside binding | 50 | 15.4321 | 2.33E-05 |
| GOTERM_MF_FAT | GO:0030554~adenyl nucleotide binding | 49 | 15.12346 | 3.34E-05 |
| GOTERM_MF_FAT | GO:0000166~nucleotide binding | 62 | 19.1358 | 6.94E-05 |
| Annotation Cluster 9 | Enrichment Score: 4.746369075698963 |  |  |  |
| Category | Term | Count | % | P Value |
| GOTERM_BP_FAT | GO:0043933~macromolecular complex subunit organization | 33 | 10.18519 | 3.43E-06 |
| GOTERM_BP_FAT | GO:0065003~macromolecular complex assembly | 31 | 9.567901 | 7.06E-06 |
| GOTERM_BP_FAT | GO:0070271~protein complex biogenesis | 25 | 7.716049 | 2.69E-05 |
| GOTERM_BP_FAT | GO:0006461~protein complex assembly | 25 | 7.716049 | 2.69E-05 |
| GOTERM_BP_FAT | GO:0051259~protein oligomerization | 13 | 4.012346 | 1.06E-04 |
| Annotation Cluster 10 | Enrichment Score: 4.496119000976713 |  |  |  |
| Category | Term | Count | % | P Value |
| GOTERM_BP_FAT | GO:0006259~DNA metabolic process | 33 | 10.18519 | 1.48E-09 |
| GOTERM_BP_FAT | GO:0006974~response to DNA damage stimulus | 19 | 5.864198 | 2.32E-04 |
| GOTERM_BP_FAT | GO:0006281~DNA repair | 16 | 4.938272 | 3.00E-04 |
| GOTERM_BP_FAT | GO:0033554~cellular response to stress | 20 | 6.17284 | 0.010074 |

**Table S8.**

Gene networks down-regulated in CTL cells relative to PE by shifting from 4 % to 20 % O_2_ culture conditions.

| Annotation Cluster 1 | Enrichment Score: 3.2635381513430946 | |  | |  | |  | |
| --- | --- | --- | --- | --- | --- | --- | --- | --- |
| Category | Term | | Count | | % | | P Value | |
| GOTERM_CC_FAT | GO:0033279~ribosomal subunit | | 9 | | 7.758621 | | 7.03E-07 | |
| GOTERM_BP_FAT | GO:0006414~translational elongation | | 8 | | 6.896552 | | 2.97E-06 | |
| GOTERM_CC_FAT | GO:0005840~ribosome | | 10 | | 8.62069 | | 3.91E-06 | |
| GOTERM_MF_FAT | GO:0003735~structural constituent of ribosome | | 8 | | 6.896552 | | 5.53E-05 | |
| GOTERM_CC_FAT | GO:0022626~cytosolic ribosome | | 6 | | 5.172414 | | 1.03E-04 | |
| GOTERM_BP_FAT | GO:0006412~translation | | 10 | | 8.62069 | | 2.07E-04 | |
| GOTERM_CC_FAT | GO:0044445~cytosolic part | | 7 | | 6.034483 | | 2.44E-04 | |
| GOTERM_CC_FAT | GO:0005829~cytosol | | 19 | | 16.37931 | | 4.53E-04 | |
| GOTERM_CC_FAT | GO:0015935~small ribosomal subunit | 5 | | 4.310345 | | 4.76E-04 | |  |
| GOTERM_CC_FAT | GO:0015934~large ribosomal subunit | | 5 | | 4.310345 | | 6.02E-04 | |
| GOTERM_CC_FAT | GO:0030529~ribonucleoprotein complex | | 11 | | 9.482759 | | 7.30E-04 | |
| GOTERM_MF_FAT | GO:0005198~structural molecule activity | | 10 | | 8.62069 | | 0.01146 | |
| GOTERM_CC_FAT | GO:0022625~cytosolic large ribosomal subunit | | 3 | | 2.586207 | | 0.020321 | |
| GOTERM_CC_FAT | GO:0022627~cytosolic small ribosomal subunit | | 3 | | 2.586207 | | 0.02238 | |
| GOTERM_MF_FAT | GO:0003723~RNA binding | | 10 | | 8.62069 | | 0.023877 | |
| GOTERM_CC_FAT | GO:0043228~non-membrane-bounded organelle | | 23 | | 19.82759 | | 0.034552 | |
| GOTERM_CC_FAT | GO:0043232~intracellular non-membrane-bounded organelle | | 23 | | 19.82759 | | 0.034552 | |
| Annotation Cluster 2 | Enrichment Score: 1.816979280238329 | |  | |  | |  | |
| Category | Term | | Count | | % | | P Value | |
| GOTERM_CC_FAT | GO:0015934~large ribosomal subunit | | 5 | | 4.310345 | | 6.02E-04 | |
| GOTERM_BP_FAT | GO:0042273~ribosomal large subunit biogenesis | | 3 | | 2.586207 | | 0.00166 | |
| GOTERM_BP_FAT | GO:0042254~ribosome biogenesis | | 3 | | 2.586207 | | 0.175358 | |
| GOTERM_BP_FAT | GO:0022613~ribonucleoprotein complex biogenesis | | 3 | | 2.586207 | | 0.307843 | |
| Annotation Cluster 3 | Enrichment Score: 1.1594855691623949 | |  | |  | |  | |
| Category | Term | | Count | | % | | P Value | |
| GOTERM_BP_FAT | GO:0051789~response to protein stimulus | | 4 | | 3.448276 | | 0.028883 | |
| GOTERM_BP_FAT | GO:0006986~response to unfolded protein | | 3 | | 2.586207 | | 0.071913 | |
| GOTERM_BP_FAT | GO:0010033~response to organic substance | | 8 | | 6.896552 | | 0.159991 | |
| Annotation Cluster 4 | Enrichment Score: 1.1408423406467503 | |  | |  | |  | |
| Category | Term | | Count | | % | | P Value | |
| GOTERM_BP_FAT | GO:0006323~DNA packaging | | 5 | | 4.310345 | | 0.006027 | |
| GOTERM_BP_FAT | GO:0006334~nucleosome assembly | | 4 | | 3.448276 | | 0.01534 | |
| GOTERM_BP_FAT | GO:0031497~chromatin assembly | | 4 | | 3.448276 | | 0.01684 | |
| GOTERM_BP_FAT | GO:0065004~protein-DNA complex assembly | | 4 | | 3.448276 | | 0.018964 | |
| GOTERM_BP_FAT | GO:0034728~nucleosome organization | | 4 | | 3.448276 | | 0.02008 | |
| GOTERM_BP_FAT | GO:0006333~chromatin assembly or disassembly | | 4 | | 3.448276 | | 0.044454 | |
| GOTERM_BP_FAT | GO:0043933~macromolecular complex subunit organization | | 8 | | 6.896552 | | 0.151384 | |
| GOTERM_BP_FAT | GO:0034621~cellular macromolecular complex subunit organization | | 5 | | 4.310345 | | 0.181346 | |
| GOTERM_BP_FAT | GO:0051276~chromosome organization | | 6 | | 5.172414 | | 0.183045 | |
| GOTERM_BP_FAT | GO:0006325~chromatin organization | | 5 | | 4.310345 | | 0.207924 | |
| GOTERM_BP_FAT | GO:0065003~macromolecular complex assembly | | 7 | | 6.034483 | | 0.231293 | |
| GOTERM_BP_FAT | GO:0034622~cellular macromolecular complex assembly | | 4 | | 3.448276 | | 0.316303 | |
| GOTERM_BP_FAT | GO:0008284~positive regulation of cell proliferation | | 3 | | 2.586207 | | 0.732638 | |
| Annotation Cluster 5 | Enrichment Score: 0.9878766195660744 | |  | |  | |  | |
| Category | Term | | Count | | % | | P Value | |
| GOTERM_CC_FAT | GO:0048770~pigment granule | | 4 | | 3.448276 | | 0.014824 | |
| GOTERM_CC_FAT | GO:0042470~melanosome | | 4 | | 3.448276 | | 0.014824 | |
| GOTERM_CC_FAT | GO:0016023~cytoplasmic membrane-bounded vesicle | | 6 | | 5.172414 | | 0.212875 | |
| GOTERM_CC_FAT | GO:0031988~membrane-bounded vesicle | | 6 | | 5.172414 | | 0.231878 | |
| GOTERM_CC_FAT | GO:0031410~cytoplasmic vesicle | | 6 | | 5.172414 | | 0.314357 | |
| GOTERM_CC_FAT | GO:0031982~vesicle | | 6 | | 5.172414 | | 0.346723 | |
| Annotation Cluster 6 | Enrichment Score: 0.8419491253380418 | |  | |  | |  | |
| Category | Term | | Count | | % | | P Value | |
| GOTERM_BP_FAT | GO:0009408~response to heat | | 3 | | 2.586207 | | 0.047275 | |
| GOTERM_BP_FAT | GO:0009266~response to temperature stimulus | | 3 | | 2.586207 | | 0.093968 | |
| GOTERM_BP_FAT | GO:0009628~response to abiotic stimulus | | 3 | | 2.586207 | | 0.670719 | |
| Annotation Cluster 7 | Enrichment Score: 0.8036657439093453 | |  | |  | |  | |
| Category | Term | | Count | | % | | P Value | |
| GOTERM_BP_FAT | GO:0001501~skeletal system development | | 5 | | 4.310345 | | 0.13665 | |
| GOTERM_BP_FAT | GO:0001503~ossification | | 3 | | 2.586207 | | 0.159958 | |
| GOTERM_BP_FAT | GO:0060348~bone development | | 3 | | 2.586207 | | 0.177578 | |
| Annotation Cluster 8 | Enrichment Score: 0.6851068413542081 | |  | |  | |  | |
| Category | Term | | Count | | % | | P Value | |
| GOTERM_CC_FAT | GO:0044432~endoplasmic reticulum part | | 6 | | 5.172414 | | 0.050449 | |
| GOTERM_CC_FAT | GO:0005783~endoplasmic reticulum | | 10 | | 8.62069 | | 0.10162 | |
| GOTERM_CC_FAT | GO:0005789~endoplasmic reticulum membrane | | 4 | | 3.448276 | | 0.204111 | |
| GOTERM_CC_FAT | GO:0042175~nuclear envelope-endoplasmic reticulum network | | 4 | | 3.448276 | | 0.226768 | |
| GOTERM_CC_FAT | GO:0012505~endomembrane system | | 7 | | 6.034483 | | 0.298808 | |
| GOTERM_CC_FAT | GO:0031090~organelle membrane | | 9 | | 7.758621 | | 0.299995 | |
| GOTERM_CC_FAT | GO:0005794~Golgi apparatus | | 5 | | 4.310345 | | 0.752435 | |
| Annotation Cluster 9 | Enrichment Score: 0.578796740243513 | |  | |  | |  | |
| Category | Term | | Count | | % | | P Value | |
| GOTERM_BP_FAT | GO:0007049~cell cycle | | 8 | | 6.896552 | | 0.206221 | |
| GOTERM_BP_FAT | GO:0022403~cell cycle phase | | 5 | | 4.310345 | | 0.25584 | |
| GOTERM_BP_FAT | GO:0022402~cell cycle process | | 6 | | 5.172414 | | 0.273762 | |
| GOTERM_BP_FAT | GO:0000279~M phase | | 4 | | 3.448276 | | 0.335072 | |
| Annotation Cluster 10 | Enrichment Score: 0.45881934904496813 | |  | |  | |  | |
| Category | Term | | Count | | % | | P Value | |
| GOTERM_BP_FAT | GO:0051259~protein oligomerization | | 4 | | 3.448276 | | 0.093913 | |
| GOTERM_BP_FAT | GO:0006916~anti-apoptosis | | 4 | | 3.448276 | | 0.136314 | |
| GOTERM_BP_FAT | GO:0043933~macromolecular complex subunit organization | | 8 | | 6.896552 | | 0.151384 | |
| GOTERM_BP_FAT | GO:0043066~negative regulation of apoptosis | | 5 | | 4.310345 | | 0.177649 | |
| GOTERM_BP_FAT | GO:0043069~negative regulation of programmed cell death | | 5 | | 4.310345 | | 0.183826 | |
| GOTERM_BP_FAT | GO:0060548~negative regulation of cell death | | 5 | | 4.310345 | | 0.18507 | |
| GOTERM_BP_FAT | GO:0065003~macromolecular complex assembly | | 7 | | 6.034483 | | 0.231293 | |
| GOTERM_BP_FAT | GO:0008219~cell death | | 6 | | 5.172414 | | 0.463805 | |
| GOTERM_BP_FAT | GO:0016265~death | | 6 | | 5.172414 | | 0.469902 | |
| GOTERM_BP_FAT | GO:0012501~programmed cell death | | 5 | | 4.310345 | | 0.52952 | |
| GOTERM_BP_FAT | GO:0042981~regulation of apoptosis | | 6 | | 5.172414 | | 0.564043 | |
| GOTERM_BP_FAT | GO:0043067~regulation of programmed cell death | | 6 | | 5.172414 | | 0.573021 | |
| GOTERM_BP_FAT | GO:0010941~regulation of cell death | | 6 | | 5.172414 | | 0.576364 | |
| GOTERM_BP_FAT | GO:0070271~protein complex biogenesis | | 4 | | 3.448276 | | 0.611885 | |
| GOTERM_BP_FAT | GO:0006461~protein complex assembly | | 4 | | 3.448276 | | 0.611885 | |
| GOTERM_BP_FAT | GO:0006915~apoptosis | | 4 | | 3.448276 | | 0.728401 | |
| GOTERM_BP_FAT | GO:0007166~cell surface receptor linked signal transduction | | 5 | | 4.310345 | | 0.998037 | |

**Table S9.**

Primers for coding sequence regions of four tested genes and the reference gene GAPDH.

| Gene | Accession # | Primer sequence | T_m_ °C | Bases | Product length (bp) |
| --- | --- | --- | --- | --- | --- |
| *HPLN 1* | NM_001884 | **F:**TGA TCC ACC CCA CCA AAC TG  **R:**CCT AGA GAT GGG GTA GCG GA | **57.5**  **57.8** | **20**  **20** | **170** |
| *CXCL1* | NM_001511 | **F:**GCA GGG AAT TCA CCC CAA GA  **R:**GAT GCA GGA TTG AGG CAA GC | **57.5**  **56.8** | **20**  **20** | **123** |
| *IL8* | NM_000584 | **F:**CCG GAA GGA ACC ATC TCA CT  **R:**AGC ACT CCT TGG CAA AAC TG | **56.6**  **56.0** | **20**  **20** | **117** |
| *MT1G* | NM_005950 | **F:**TCT CGC CTC GGG TTG CAA TG  **R:**CTA TTT GTA CTT GGG AGC AGG GC | **60.4**  **57.7** | **20**  **23** | **236** |
| *MT1G* | NM_005950 | **F:**AGG CTG CAT CTG CAA AGG GG  **R:**AGC AAA GGG GTC AAG ATT GTA GC | **60.7**  **57.5** | **20**  **23** | **139** |
| *GAPDH* | NM_002046 | **F:**CTG GGG CTG GCA TTG CCC TC  **R:**GGC AGG GAC TCC CCA GCA GT | **64.0**  **64.5** | **20**  **20** | **192** |
